# Nano-Confined Solar-Thermal Water Purification Boosted by Physical Field Disturbance Coupled with Ultrafast Non-Radical Advanced Oxidation Process

**DOI:** 10.1007/s40820-026-02249-x

**Published:** 2026-07-06

**Authors:** Fan-Zhen Jiao, Xiaoyang Fang, Sheng-Xing Hou, Zhi-Hao Wang, Wenbo You, Zhenzhong Yang, Zhong-Zhen Yu, Jin Qu

**Affiliations:** 1 State Key Laboratory of Organic−Inorganic Composites, Beijing University of Chemical Technology, Beijing, 100029 People’s Republic of China; 2 Center for Nanomaterials and Nanocomposites, College of Materials Science and Engineering, Beijing University of Chemical Technology, Beijing, 100029 People’s Republic of China; 3 Shanghai Key Laboratory of Atmospheric Particle Pollution and Prevention, Department of Environmental Science and Engineering, Fudan University, Shanghai, 200433 People’s Republic of China; 4 Department of Chemical Engineering, Tsinghua University, Beijing, 100084 People’s Republic of China

**Keywords:** Solar-thermal water evaporation, Non-radical advanced oxidation process, Physical field disturbance, Electron transfer pathway, Nano-confinement effect

## Abstract

Solar interfacial evaporation has undergone rapid development in recent years, yet its overall performance has reached a plateau due to limited advances in solar-thermal materials. Herein, we propose a synergistic nano-confinement and physical-field–modulation strategy that enables concurrent acceleration of solar-driven evaporation of water and on-site remediation of organic pollutants. Implemented in hollow mesoporous carbon nanocages integrated with Fe–N_4_ catalytic sites and inner wall plasmonic Au nanoparticles, the system couples mesoporous confinement with localized thermal and pressure perturbations to transform bulk water into thermodynamically activated intermediate states and substantially reduce the effective vaporization enthalpy. This integrated framework delivers high evaporation rates of 2.56 kg m^−2^ h^−1^ in planar devices and 6.84 kg m^−2^ h^−1^ in 3D architectures under one-sun irradiation, with a kinetic enhanced Hertz–Knudsen–Schrage–derived evaporation coefficient. Simultaneously, the Fe–N_4_ sites enable non-radical peroxymonosulfate activation for ultrafast degradation of bisphenol A, achieving a rate of 182.5 L g^−1^ min^−1^. This work establishes an ingenious strategy for coupling water-state regulation and catalytic pollutant degradation to break the performance bottleneck of solar-thermal purification.

**Supplementary Information:**

The online version contains supplementary material available at 10.1007/s40820-026-02249-x.

## Highlights

- Synergistic nano-confinement and physical field modulation thermodynamically activate water and accelerate solar-driven evaporation, achieving high rates up to 6.84 kg m^−2^ h^−1^ under one-sun irradiation.
- The hollow nanocage architecture with highly accessible Fe–N_4_ sites enables non-radical peroxymonosulfate activation, delivering ultrafast bisphenol A degradation with a mass-normalized rate constant of 182.5 L g^−1^ min^−1^.
- Concurrent acceleration of solar-driven evaporation of water and on-site remediation of organic pollutants is achieved.

**Supplementary Information:**

The online version contains supplementary material available at 10.1007/s40820-026-02249-x.

## Graphical abstract

**Figure:**
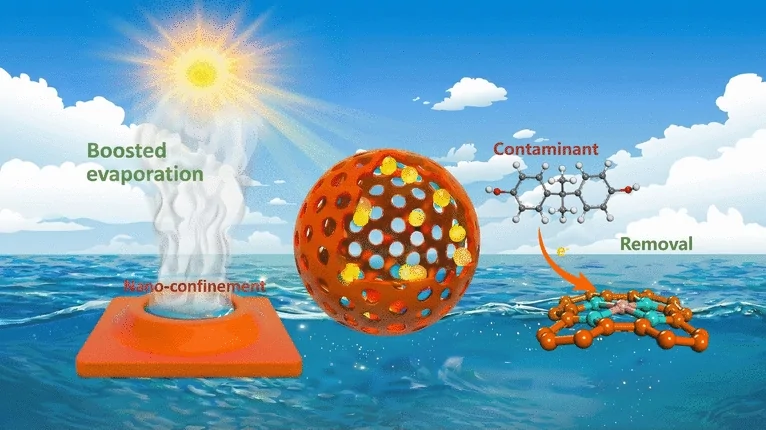

## Introduction

Clean and safe freshwater is the cornerstone for human survival, socio-economic development, and ecological stability, making it one of the most crucial resources nowadays [1, 2]. In recent years, solar-driven interfacial evaporation has attracted considerable attentions as an approach to mitigate freshwater scarcity, particularly in the regions with limited access to clean water [3–5]. Many types of solar-thermal evaporators have been developed by using diverse solar-thermal materials and hydrophilic substrates, offering distinct advantages such as the reduction of water vaporization enthalpy (ΔHevp), effective utilization of solar and ambient energy, structural flexibility, and high mechanical stability [6–10]. However, different from the evaporation thermodynamics activity and mechanical properties, the evaporation kinetic activity of solar-thermal materials is rarely taken into consideration. The heat generation and transfer, and the water diffusion and transport are crucial for realizing efficient solar steam generation of solar-driven interfacial evaporators. Therefore, the accurate control in terms of directional transfer and diffusion of both heat and water at the nanoscale is particularly vital for maximizing solar-driven interfacial evaporation and designing new generation solar-thermal interfacial evaporators. In addition, the most solar-thermal evaporation systems are less effective in purifying seawater/wastewater containing complex organic pollutants. To fully address the water pollution issues, it is therefore highly promising to integrate efficient catalytic degradation of organic pollutants with fast solar-thermal evaporation of seawater/wastewater. Previously, catalytic degradation of pollutants and solar-thermal evaporation of water are usually treated as separate processes, where catalysis is merely implemented by coating or in situ growth of catalysts onto porous evaporation substrates [11–14], causing additional structure fabrication and inevitable performance conflicts. Clearly, the design of new generation solar-thermal evaporators that integrate enhanced water evaporation kinetic activity at the nanoscale with fast catalytic degradation of organic pollutants is requisite for efficient solar-driven water purification.

**Fig. 1:**
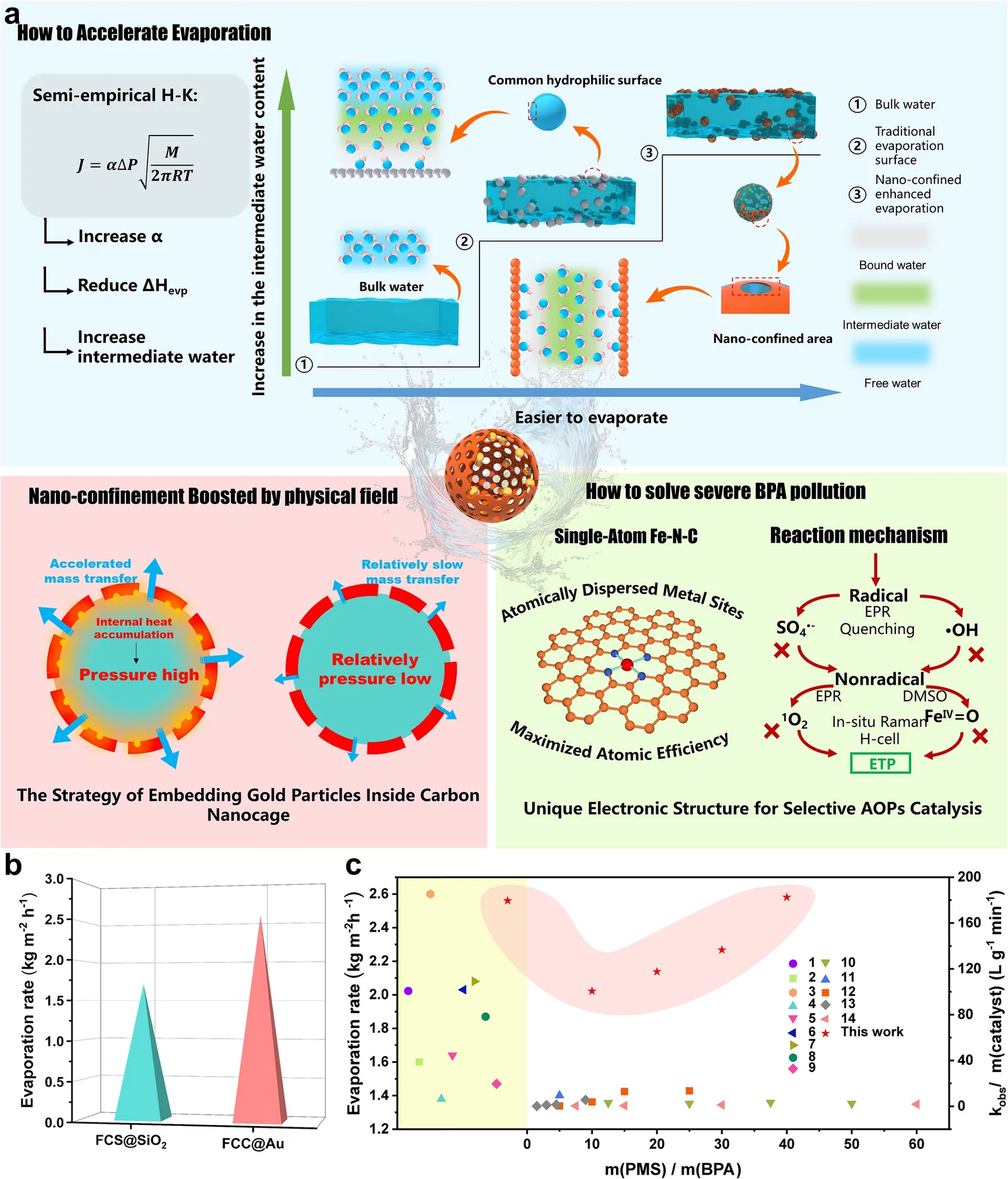
**a** Schematic diagram of evaporation-promoting principle of nano-confined structure and how Fe–N–C sites catalyze the degradation of BPA. **b** Comparison of evaporation rates of 2D FCC@Au evaporator with nano-confinement enhancement and 2D FCS@SiO_2_ evaporator without such an enhancement. **c** The yellow area comparing the evaporation performance of the 2D FCC@Au evaporator with other reported 2D evaporators, and the white area comparing the BPA degradation performances of FCC@Au compared with other catalysts reported

The interface temperature and the vaporization enthalpy (ΔHevp) are taken as important indicators for confirming thermodynamic activity of the interfacial evaporation. Generally, the interfacial evaporation rate can be described by the Hertz–Knudsen–Schrage relation [15–17]:

1 $$J = \frac{2\alpha }{{2 - \alpha }}\sqrt {\frac{M}{{2{\uppi }RT_{i} }}} \left( {p_{{{\mathrm{sat}}}} \left( {T_{i} } \right) - p_{v} } \right)$$

where *J* is the mass flux, *α* is the kinetic accommodation (evaporation) coefficient, *M* is the molar mass of water, *R* is the universal gas constant, *T*_*i*_ is the interface temperature, *p*_sat_(*T*_*i*_) is the saturation vapor pressure at *T*_*i*_, and *p*_*v*_ is the ambient vapor pressure. In a solar-thermal evaporation system, although the increase in *T*_*i*_ can raise *p*_sat_ and thus the flux, a more effective strategy is required to directly enhance the evaporation coefficient α, facilitating the detachment of water molecules from the liquid phase. Fortunately, nanoscale confinement provides a practical means to achieve fast water evaporation. As shown in Fig. 1a**②**, when water molecules come into contact with the surface of a hydrophilic material, they can be divided into three types: bound water that is closest to and has strong interactions with the surface of the material; weakly bound water with moderate distances from the surface of the material where the hydrogen bonding network of the water is disrupted, which is known as intermediate water; and free water far away from the surface of the material that is almost unaffected by the material with intact hydrogen bonding network [18]. When water is confined within nanopores, nanotubes, or layered channels, the surface effect is amplified and more intermediate water can be reorganized (Fig. 1a**③**), improving diffusion, dielectric response, and solvation behavior [19–21]. In addition, the curvature effect (Kelvin effect) ascribed from the nanopores can enhance the thermodynamic driving force further [22–24].

Inspired by this, if there are physical fields to continuously pump the water through a nano-confined pore, the distance limitation facilitates the constant generation of more intermediate water that is easier to be evaporated than bound water and free water, significantly increasing α (Fig. 1a). Therefore, artificial guiding water with constructed physical fields through nano-confined pathways can not only leverage solar-thermal heating to elevate the interfacial temperature but, more importantly, modulate water molecular structure to increase the evaporation coefficient, thereby boosting the water evaporation substantially.

Advanced oxidation processes (AOPs) have been proven to be effective in purifying wastewater bodies with complex and diverse compositions, such as biorefractory and toxic organic pollutants [25, 26]. Especially, atomically dispersed single-atom catalysts (SACs) usually have high metal site exposure and atom utilization, and their strong metal-N bonds can keep themselves structurally stable and work well under harsh conditions, such as seawater environment [27]. Unlike radical-based advanced oxidation processes that proceed via indiscriminate attacks, SACs usually exhibit non-radical catalytic processes including the electron transfer pathway (ETP), which can therefore achieve notable selectivity and is more advantageous than radical-based advanced oxidation processes. The oxidation efficiency of ETP is mainly governed by the alignment between the highest occupied molecular orbital (HOMO) of a pollutant and the lowest unoccupied molecular orbital (LUMO) of the oxidant. Therefore, ETP is well suitable for electron-rich compounds, such as phenols and aromatic amines. Additionally, ETP is less susceptible to inorganic ions (e.g., Cl^−^ and HCO_3_^−^) and natural organic matters, thus enabling more targeted oxidation in complex aqueous environments. Very recently, extensive efforts have been devoted to exploring ETP in single-atom metal-referenced carbon-based catalysts [25, 26, 28]. As potential solar-thermal materials, carbon-based SACs can also accelerate their own catalytic processes via their solar-thermal effect. Currently, however, there are no reports on nanoporous carbon-based SACs that achieve simultaneous enhancements in solar-thermal water evaporation and ETP-based AOPs catalysis driven by physical fields.

Herein, we report a rational design of nitrogen-coordinated single-atom Fe-doped mesoporous carbon nanocages encapsulating plasmonic Au nanoparticles on their inner walls (FCC@Au) for efficiently purifying wastewater by simultaneous solar-thermal evaporation and non-radical catalysis. The hollow carbon architecture synergistically integrates broadband solar light harvesting of the carbon component, high thermodynamic activity of water ascribed to the nano-confinement effect of the mesoporous carbon shells, and promoted evaporation kinetic activity attributed to the localized physical field modulation from the interior of the nanocage to the exterior of the shell induced by the solar irradiation of the Au nanoparticles embedded on the inner surface of the nanocage, thereby promoting the generation of intermediate water, reducing the water vaporization enthalpy, promoting the water evaporation coefficient, and thus achieving an ultrafast water evaporation rate of 2.56 kg m^−2^ h^−1^, which is one of the highest values reported for 2D planar solar-thermal evaporators (Fig. 1b). Beyond the water evaporation, the atomically dispersed Fe–N–C sites act as highly accessible and structurally robust catalytic centers that drive non-radical electron transfer pathways with pronounced selectivity, while the hollow configuration further amplifies catalytic turnover by offering an ultrahigh surface area, yielding a mass-normalized rate constant of 182.5 L g^−1^ min^−1^, which is nearly two orders of magnitude higher than most catalysts reported (Fig. 1c, Table S1). By intrinsically coupling efficient solar-thermal evaporation with advanced oxidation, the design of FCC@Au exemplifies a new paradigm for sustainable and efficient purification of seawater/wastewaters containing complex organic pollutants.

## Experimental Section

### Chemicals

Dopamine hydrochloride, tris(hydroxymethyl)aminomethane (Tris), hemin chloride, and tetraethyl orthosilicate (TEOS) were purchased from Aladdin (China). Ethanol, ammonia solution (25%), dimethyl sulfoxide (DMSO), sodium hydroxide, and hydrochloric acid (12 M) were obtained from Fuyu Chemical Co., Ltd. Aqueous chloroauric acid solution (23.5%–23.8%), 3-aminopropyltriethoxysilane (KH-550), anhydrous potassium carbonate, potassium citrate, polyvinyl alcohol (PVA, 1799), and glutaraldehyde solution (25%) were supplied by Macklin (China). Sodium borohydride was purchased from Alfa Aesar. Anhydrous sodium sulfate, and bisphenol A (BPA) were obtained from China National Pharmaceutical Group. 5,5-Dimethyl-1-pyrroline N-oxide (DMPO), 2,2,6,6-tetramethyl-4-piperidinol (TEMP), and potassium peroxymonosulfate (PMS) were purchased from Sigma-Aldrich.

### Synthesis of Silica Microsphere Template

Uniform silica microspheres were synthesized via the Stöber method. Briefly, 6 mL of tetraethyl orthosilicate was added to a mixture of 75 mL ethanol and 10 mL deionized water under stirring. Then, 3.5 mL of ammonia solution was added, and the mixture was allowed to react for 1 h. The resulting product was collected by centrifugation, washed thoroughly, and dried to obtain silica microsphere templates.

### Synthesis of Gold Nanoparticle-Loaded Silica Templates

Gold nanoparticles were deposited on the silica spheres with an amine-mediated surface modification method. Specifically, 1 g of silica microspheres were ultrasonically dispersed in 150 mL ethanol, followed by the addition of 5 mL 3-aminopropyltriethoxysilane. After stirring for 2 h, the amino-functionalized silica was collected by centrifugation, washed, and stored in ethanol. Subsequently, 20 mL of 0.1 M NaOH was added to 100 mL of 6.35 mM aqueous HAuCl₄ solution and stirred for 30 min. Then, 500 mg of amino-functionalized silica spheres were added, and the mixture was reacted at 75 °C for 1 h. The resulting orange-colored dispersion was centrifuged, washed, and re-dispersed in 100 mL deionized water to obtain Au seed-loaded silica microspheres.

The growth of gold nanoparticles was conducted in a potassium–gold solution (K-gold). K-gold was prepared by dissolving 30 mL of 6.35 mM HAuCl₄ and 300 mg of potassium carbonate in 500 mL deionized water and aging the mixture overnight at 25 °C in the dark. On the next day, 100 mL of Au seed-loaded silica was added to the K-gold solution and stirred for 15 min. Then, 16.7 mg of NaBH₄ dissolved in 10 mL of ice-cold water was introduced, and the reaction proceeded for 20 min. Finally, 25 mL of 0.1 mM sodium citrate was added to quench the reaction. The product was collected by centrifugation, washed, and dried to obtain gold nanoparticle-loaded silica microsphere templates.

### Synthesis of N-Coordinated Single-Atom Fe-Doped Hollow Carbon Nanocages

The carbon nanocages were prepared via a polymer coating–pyrolysis–etching strategy. For single-atom Fe doping, hemin chloride and dopamine hydrochloride were co-polymerized during the polymer coating step. Typically, 1 g of gold-loaded silica microspheres were ultrasonically dispersed in 150 mL deionized water. Separately, 500 mg of Tris was dissolved in 20 mL deionized water, followed by the addition of 50 mg hemin chloride to form solution A. Meanwhile, 750 mg of dopamine hydrochloride was dissolved in 30 mL deionized water as solution B. Solutions A and B were then added to the silica dispersion and stirred for 6 h. The product was collected by centrifugation, washed, and dried to obtain the hemin/polydopamine-coated precursor.

The dried precursor was pyrolyzed in a tubular furnace at 900 °C for 2 h in an argon atmosphere with a heating rate of 5 °C min^−1^. After natural cooling, the pyrolyzed product was etched with 4 M NaOH at 50 °C for 48 h, followed by a secondary washing step using 2.4 M HCl to remove excess iron clusters. The final product was denoted as hollow Fe single-atom doped carbon nanocages encapsulating gold nanoparticles (FCC@Au). For comparison, three control samples were also prepared. A non-etched sample was denoted as FCS@Au. When pure SiO_2_ microspheres were used as the template instead of gold-loaded ones, the resulting material was labeled FCC. In addition, a sample synthesized without the addition of hemin chloride during the polymerization step was referred to as NCC.

### Characterization

The morphology and microstructure of the samples were characterized using a FEI Talos F200S high-resolution transmission electron microscope (HRTEM), a HITACHI SU8600 scanning electron microscope (SEM), and a Thermo Fisher Titan Themis G2 300 high-angle annular dark-field scanning transmission electron microscopy (HAADF-STEM). X-ray diffraction (XRD) patterns were collected on a RIGAKU Ultima IV diffractometer equipped with a Cu K*α* radiation source at a scan rate of 5° min^−1^. Fe K-edge X-ray absorption near-edge structure (XANES) spectra were measured at the BL14W1 beamline of the Shanghai Synchrotron Radiation Facility (SSRF) in transmission mode over an energy range of 7100–7200 eV. The samples were prepared as pelletized powders, and a Si(111) monochromator was used for energy selection. Data processing and normalization were performed using the Demeter software package [29]. X-ray photoelectron spectroscopy (XPS) analysis was conducted on a Thermo Scientific K-Alpha spectrometer. Specific surface area and pore size distribution were measured by using the Brunauer–Emmett–Teller (BET) method with a Micromeritics ASAP 2460 nitrogen adsorption analyzer. The light absorption properties were evaluated with a PerkinElmer Lambda 950 UV–vis-NIR spectrophotometer. Raman spectra were recorded on a Horiba LabRAM HR Evolution spectrometer.

### Evaluation of Solar-Thermal Conversion and Water Evaporation Performances

To prepare the 2D solar-thermal test samples, 10 mg of powdery sample was ultrasonically dispersed in 500 μL deionized water and drop-cast evenly onto a 1.5 cm diameter glass fiber membrane. After the sample was uniformly adhered to the membrane surface by filtration, the resultant membrane was placed on a polystyrene foam board that was positioned on the top of a beaker. A cotton thread was used to upward deliver water from the bottom water reservoir to the membrane surface by capillary action.

For the 3D solar-driven water evaporator configuration, the powdery sample was loaded onto a superabsorbent polyurethane foam. Specifically, 20 mg of powder was ultrasonically dispersed in 2 mL of deionized water and then dropwise applied onto the surface of the polyurethane foam. Subsequently, an aqueous solution with 3 wt% of polyvinyl alcohol was sprayed on the surface. The resulting foam was immersed in an aqueous solution with 25 wt% of glutaraldehyde (pH = 3) for 2 h to induce crosslinking. Subsequently, the foam was soaked in deionized water to remove excess glutaraldehyde.

During the performance tests, a CEL-HXUV300 xenon lamp equipped with an AM 1.5G filter was used to simulate sunlight. The light intensity was adjusted by tuning the current between 14 and 16 A under a constant voltage of 14 V, and calibrated using a CEL solar power meter. The mass losses during the solar-thermal evaporation were recorded using an Ohaus CP214 electronic balance connected to a computer. The surface temperature of the evaporator was recorded with a FLIR E40 infrared thermal imaging camera. The water evaporation rate (*v*) is calculated using Eq. (2):

2 $$v = \frac{{{\Delta }m}}{{S \cdot {\Delta }t}}$$

where *m* is the mass of water evaporation, *S* is the projective area of a sample directly exposed to the simulated sunlight, and* t* is the exposure time.

### Equivalent Evaporation Enthalpy Measurement

The equivalent evaporation enthalpy was measured on the basis of the assumption that the evaporator and pure water obtain the same energy. The vaporization enthalpy of pure water can be obtained using the Eq. (3).

3 $$\Delta H{\mathrm{vap}}\left( T \right) = 2501 - 2.361 \times \left( {T - 273.15} \right)\left( {{\mathrm{kJ/kg}}} \right)$$

Therefore, the equivalent vaporization enthalpy of the evaporator can be calculated using the Eq. (4):

4 $$\Delta H{\mathrm{equ}} \times \Delta m = \Delta H\;{\mathrm{water}} \times \Delta m\;{\mathrm{water}}$$

It is worth noting that the evaporator must be a flat evaporator with the same evaporation area as that of pure water, and both are placed in the same constant temperature and humidity chamber.

### Evaluation of Catalytic Degradation Performances

The catalytic activity of the powder samples was evaluated with BPA as a model pollutant. In a typical experiment, 1 mg of catalyst was added into 50 mL of BPA solution and ultrasonically dispersed for 30 s. After the mixture was stirred at 25 °C for 30 min to reach an adsorption equilibrium, 20 mg of PMS was added to initiate the catalytic reaction. At predetermined time intervals, 1.5 mL aliquots were extracted, filtered through 0.22 μm membranes, and immediately mixed with an equal volume of ethanol to quench residual reactive species. Radical scavenging experiments were carried out by adding ethanol, TEMP, or DMSO into the system based on the original conditions. The BPA concentration was determined by a high-performance liquid chromatography (HPLC) equipped with a C18 column, using a mixture of water and methanol (v/v = 3:7) as the mobile phase. The degradation efficiency (DE) and the apparent rate constant (K_obs_) are calculated using the Eqs. (5) and (6):

5 $${\mathrm{DE}} =(1- \frac{{C_{t} }}{{C_{0} }}) \times 100\%$$

6 $$k_{{{\mathrm{obs}}}} \cdot t = - \ln \left( {\frac{{C_{t} }}{{C_{0} }}} \right)$$

where *C*_0_ is the initial concentration of BPA, and *C*_*t*_ is the concentration at time* t*.

### Electrochemical Measurements

To prepare the working electrode, 5 mg of the sample was ultrasonically dispersed in a mixture of 900 μL ethanol and 100 μL of 5 wt% Nafion solution for 1 h. Then, 50 μL of the resulting ink was drop-cast onto a glassy carbon electrode, and dried under ambient conditions. The prepared electrode was immersed in 50 mM Na_2_SO_4_ electrolyte overnight to stabilize the surface potential. Electrochemical tests were conducted using a standard three-electrode configuration, with a platinum plate as the counter electrode and an Ag/AgCl electrode as the reference electrode. Open-circuit potential (OCP) measurements were performed using a CHI-760E electrochemical workstation. Once the OCP stabilized, PMS was added to the electrolyte. After the potential reached a new stable value, BPA was introduced to initiate the reaction.

### H-Cell Oxidation Experiments

To verify that the electron transfer process proceeds via direct electron donation from BPA to the catalyst and then to PMS, a two-chamber H-type electrochemical cell was employed. A proton exchange membrane was used to separate the two chambers, which were, respectively, filled with BPA and PMS solutions. The two chambers were connected only via the catalyst-coated electrodes and an external circuit for monitoring the concentration changes and the current flow. Specifically, 10 mg of catalyst was ultrasonically dispersed in a mixture of 40 μL Nafion solution and 1 mL isopropanol for 1 h to form a homogeneous ink. Then, 0.2 mL of the ink was drop-cast onto a 2 × 2 cm^2^ piece of carbon paper and dried to prepare the working electrode.

The two compartments of the H-cell were separated using a Nafion membrane. Each chamber was filled with 50 mL of 50 mM Na₂SO₄ solution; one chamber also contained 10 ppm BPA. Identical electrodes were placed in each chamber and connected in series with an ammeter using conductive wires. PMS was added to the chamber that initially contained only Na_2_SO_4_ solution to trigger the oxidation reaction, and the resulting current change was recorded.

## Results and Discussion

### Hierarchical Structure Design and Microstructures of FCC@Au Nanocages

**Fig. 2:**
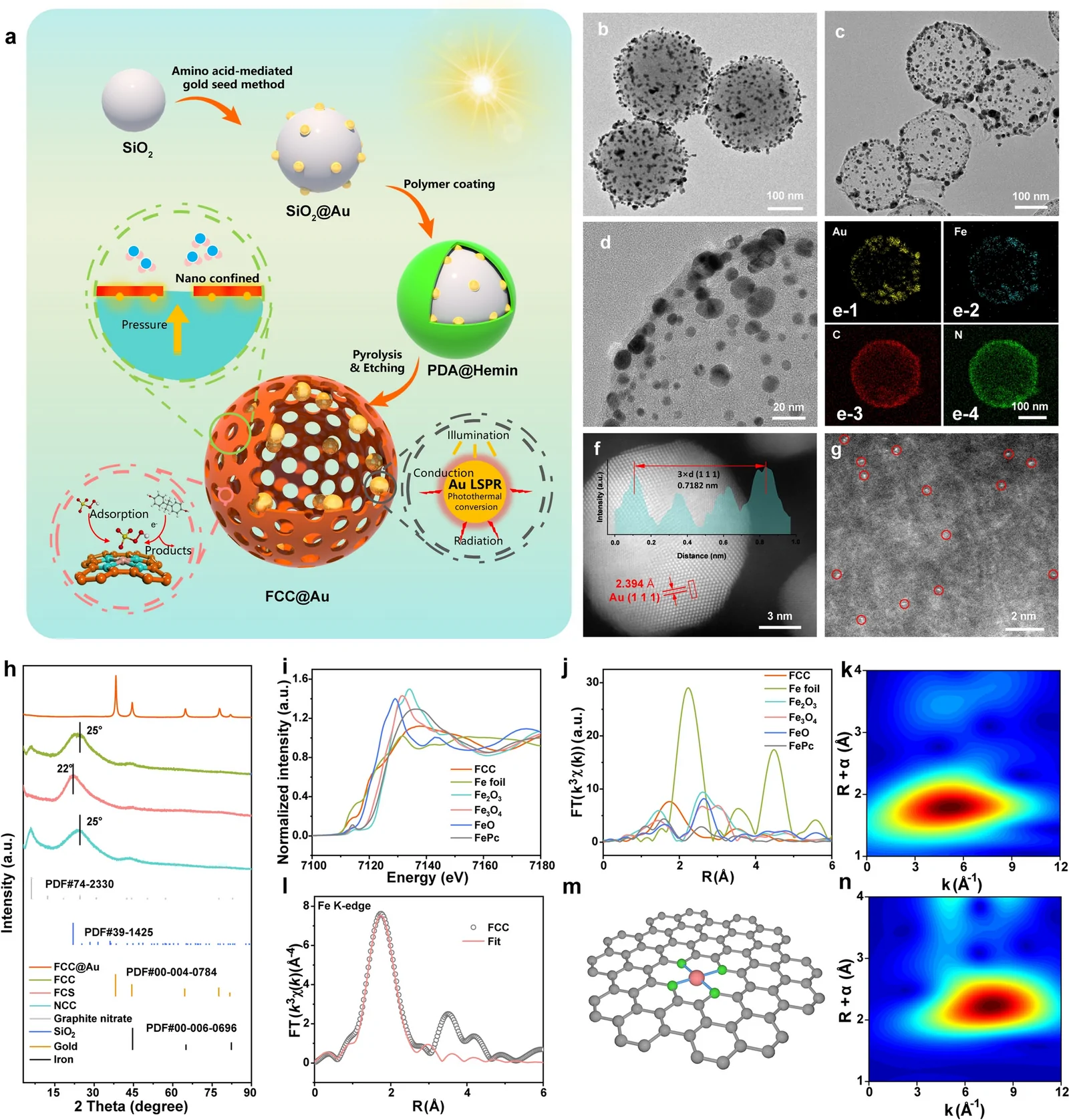
**a** Schematic illustrating the preparation of FCC@Au nanocages and Schematic diagram of efficient solar-thermal evaporation and catalytic degradation by FCC@Au. **b** HR-TEM image of SiO_2_@Au. **c**,** d** HR-TEM image of FCC@Au. **e** EDS mapping of Au, Fe, C, and N of FCC@Au. **f**,** g** HAADF-STEM image of FCC@Au, and its line scan measured along the selected region. **h** XRD patterns of FCC@Au, FCC, FCS, NCC, and standard cards of graphite nitrate, SiO_2_, Au, and Fe. **i** Fe K-edge XANES spectra, and **j** FT-EXAFS spectra of FCC and reference samples. **l** EXAFS fitting curve of FCC at Fe K-edge. **m** Schematic illustration of Fe–N_4_ coordination configuration embedded in carbon matrix. Wavelet transform contour plots of Fe K-edge EXAFS spectra for **k** FCC, and **n** Fe foil

Figure 2a schematically illustrates the rationally designed hierarchical structure of nitrogen-coordinated single-atom Fe-doped porous carbon nanocages embedded with Au nanoparticles (FCC@Au), integrating multiple functional features. The surface hydrophilic modification combined with capillary effects facilitates rapid infiltration of water into the porous carbonaceous nanocages. The embedded Au nanoparticles enhance solar-thermal energy conversion via the localized surface plasmon resonance (LSPR) to generate heat inside the nanocages. The generated thermal gradient and pressure gradient offer directional driving forces for water transport across the nano-confined porous channels. Moreover, the confined nanoscale environment facilitates the formation of more intermediate water with weakened hydrogen bonding, thereby lowering the water vaporization enthalpy. Overall, the multifunctional FCC@Au nanocages can simultaneously (i) broaden solar light absorption and enhance solar-thermal conversion efficiency, (ii) tune water molecular state for optimizing evaporation thermodynamics and kinetics, and (iii) activate PMS for efficient catalytic degradation of pollutants, which are thus highly efficient in solar-driven water evaporation while addressing water purification challenges.

FCC@Au is synthesized by sequentially coating silica spheres with Au nanoparticles (AuNPs) and a polymer layer, followed by pyrolysis at 900 °C and etching away the silica spheres (Fig. 2a). Hemin chloride is introduced as the Fe precursor during the polymer coating, enabling the formation of atomically dispersed Fe sites via high-temperature pyrolysis in an argon atmosphere. The hierarchical structure features of FCC@Au are verified by a high-resolution transmission electron microscopy (HR-TEM, Fig. 2b–d), and compared with those of its counterpart (FCC) without Au nanoparticles (Fig. S1). Apparently, the carbonaceous nanocages exhibit a uniform monodisperse morphology with an average diameter of ~ 200 nm, and the Au nanoparticles are homogeneously distributed inside. The corresponding elemental mapping images (Fig. 2e, panels 1–4) confirm the uniform distributions of Fe and N species across the carbon shells. It should be noted that the apparent spatial overlap between Fe and Au signals in the EDS mapping arises from a projection effect of the hollow nanocage structure in TEM observation, rather than from Fe–Au alloying or direct chemical bonding. The high-resolution lattice imaging (Fig. 2f) identifies distinct crystallographic planes of Au nanoparticles with an interplanar spacing of 2.39 Å, consistent with the (1 1 1) facet of the face-centered cubic gold, which is the thermodynamically favored surface of Au nanocrystals. Furthermore, the aberration-corrected high-angle annular dark-field scanning transmission electron microscopy (HAADF-STEM) image (Fig. 2g) clearly demonstrates the atomically dispersed Fe sites anchored in the carbon shells.

To further verify the synthesized hierarchical structure, Fig. 2h shows X-ray diffraction (XRD) pattern of FCC@Au, nitrogen-coordinated single-atom Fe-doped carbon nanocages (FCC), nitrogen-coordinated single-atom Fe-doped carbon nanospheres (FCS), and N doped carbon nanocages NCC. FCC@Au presents sharp diffraction peaks attributed to the high crystallinity of metallic Au, which dominates the pattern and weakens the signals from other components. These peaks are consistent with the standard card of the elemental gold, achieving the loading of Au nanoparticles. The carbon peak has not truly disappeared; it is just too small compared to the relatively strong gold peak. In Fig. S2, by adjusting the scale range for the corresponding carbon peak, a broad amorphous carbon diffraction peak can still be observed. In comparison, FCC, FCS, and NCC display broad diffraction humps in the range of 20°–30°, which is ascribed to the overlap between the primary diffraction signals of amorphous carbon and residual silica. In particular, FCC and NCC primarily exhibit the carbon peak at ~ 25°, while FCS has a peak near 22°, closer to that of SiO_2_ (Fig. S3). A low-angle diffraction signal, associated with the graphitic carbon nitride, is present in all samples, verifying the effective doping of nitrogen.

Furthermore, the absence of any diffraction peaks of metallic Fe suggests that no Fe clusters are formed during the synthesis. X-ray absorption near-edge structure (XANES) spectroscopy is employed to elucidate the oxidation state and coordination environment of Fe species in FCC, in which the Fe content is determined to be 0.77 wt% by ICP-OES analysis [30]. As shown in Fig. 2i, the Fe K-edge absorption edge of FCC lies in between those of Fe foil (Fe^0^) and Fe_2_O_3_ (Fe^3^⁺), but is closer to that of FePc (Fe^2^⁺), demonstrating that Fe in FCC predominantly exists in its + 2 oxidation state. The relatively lower white line intensity compared to Fe_2_O_3_ further supports a less oxidized Fe center with partially filled 3d orbitals. Moreover, the enhanced pre-edge feature indicates increased local structural distortion and stronger 3d–4p orbital hybridization around the Fe center, consistent with the formation of a low-coordinate planar Fe–N₄ configuration. Such a coordination configuration promotes efficient electron transfer by lowering the activation barrier for PMS activation.

Further structural insights into the local coordination of Fe atoms are explored with the Fe K-edge extended an X-ray absorption fine structure (EXAFS) spectroscopy. The Fourier-transformed EXAFS spectra (Fig. 2j) show that the main peak of FCC appears at ~ 1.75 Å, clearly distinct from that of Fe foil (~ 2.2 Å, Fe–Fe coordination) and iron oxides (~ 1.8–2.9 Å, Fe–O/Fe coordination), verifying the isolated dispersion of Fe atoms rather than the formation of clusters or oxides. As displayed in Fig. 2l, the EXAFS fitting of FCC reveals only a single dominant coordination shell, and the fitted curve aligns closely with the experimental data. The EXAFS fitting curve of FCC at k space and the Re(k^3^χ(k)) oscillation curve are shown in Figs. S4 and S5, respectively. The fitting suggests that Fe is coordinated with four nitrogen atoms, with a bond distance of ~ 2.0 Å. The absence of Fe–Fe signal confirms the atomic dispersion of Fe species (Table S2). The structural model in Fig. 2m shows the Fe center is stabilized in a planar Fe–N_4_ coordination configuration within a graphitized carbon matrix, exhibiting D_4_h symmetry. This well-defined coordination environment is conducive to efficient electron transfer through optimized orbital overlap and charge delocalization. The wavelet transform (WT) analysis of the EXAFS spectra offers further evidence of the atomic dispersion of Fe. As seen in Fig. 2k, the WT contour of FCC shows a single scattering feature at ~ 1.7 Å and has no signals in the higher-R region, indicating the absence of heavy-element coordination such as Fe–Fe. By contrast, the WT spectra of Fe foil (Fig. 2n), Fe_2_O_3_, and Fe_3_O_4_ (Fig. S6) display complex multi-cage scattering patterns, characteristic of clustered or oxide-like Fe environments.

The chemical states of nitrogen species are examined by using the X-ray photoelectron spectroscopy (XPS), revealing further insights into the bonding environment. As shown in Fig. S7, the N 1*s* spectrum can be deconvoluted into peaks corresponding to pyridinic N (~ 398.5 eV), pyrrolic N (~ 400.5 eV), graphitic N (~ 403 eV), and oxidized N species at higher binding energies [26]. Upon the incorporation of Fe, the relative content of pyrrolic N decreases from 0.73 to 0.61, while that of pyridinic N increases from 0.16 to 0.23, suggesting the formation of Fe–N coordination bonds. Meanwhile, the graphitic N content is also elevated, likely promoted by the catalytic role of Fe during carbonization. Post-alkaline treatment further increases the proportion of oxygen-containing functional groups on the carbon surface (Fig. S8), which enhances the material’s hydrophilicity and affinity toward aqueous environments.

### Solar-Thermal Water Evaporation Properties of FCC@Au

The carbonization endows FCC with a quasi-continuous energy band structure arising from π-electron delocalization, thereby significantly enhancing its broadband light harvesting capability to ~ 95%. Benefiting from the localized surface plasmon resonance (LSPR) effect of metallic nanoparticles, the incorporation of Au particles significantly enhances the light harvesting capability of SiO_2_, improving the solar-thermal performance of FCC@Au, particularly in the visible region (Figs. S9 and S10). Consequently, the Au nanoparticles embedded within the carbon nanocages effectively promote internal solar-thermal conversion, enabling FCC@Au to reach 76 °C within one min under 1-sun irradiation (Fig. S11). The enhanced internal solar-thermal conversion promotes water vapor transport from the interior to the exterior, while the nanoscale confinement imposed by the porous nanocage (the pore size distribution and specific surface area measured with the BET method are shown in Fig. S12) accelerates the water evaporation process.

**Fig. 3:**
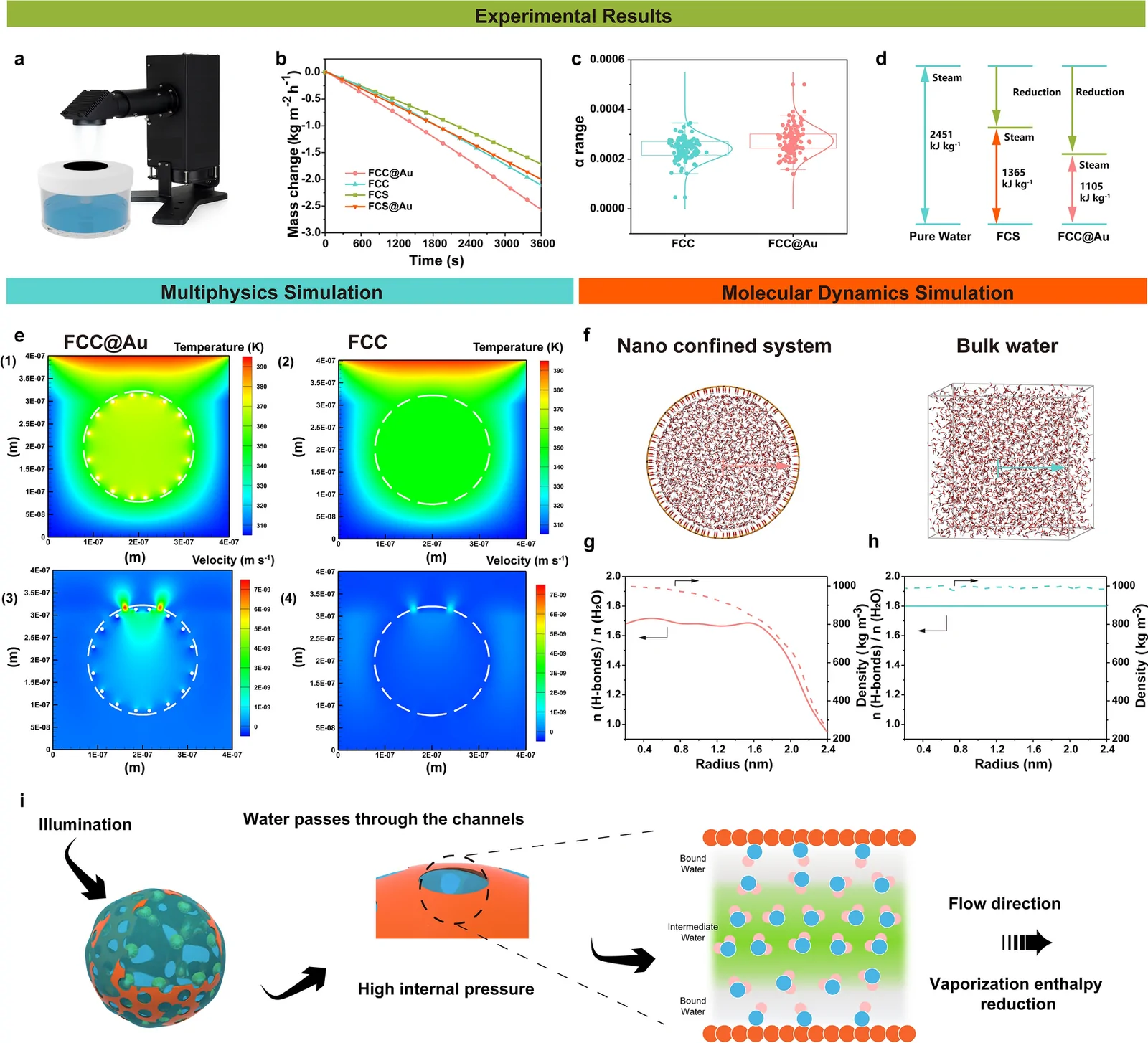
**a** Schematic diagram of a planar evaporator device made from powdery solar-thermal material. **b** Plots of water mass change of FCC@Au, FCC, FCS@Au, and FCS evaporators under 1-sun irradiation. **c** Box plots showing the statistical distribution of the calculated evaporation coefficient within 3000s. **d** Equivalent vaporization enthalpies of FCC@Au, FCS, and pure water under dark condition. **e 1**, **2** Simulated temperature distributions of FCC, and FCC@Au nanocages during solar-driven water evaporation. **e 3**,** 4** Simulated vapor pressure and velocity vector fields of FCC, and FCC@Au nanocages. **f** Final snapshot from MD simulations showing bulk water, and the water in confined nanopores. Radial distributions of average hydrogen bond number and water density derived from MD simulation **g** in nano-confined system, and **h** bulk water. **i** Schematic diagram of the effect of FCC@Au on promoting evaporation after being exposed to light due to the physical field disturbance boosting nano-confinement

As shown in Fig. 3a, b, the planar evaporation tests by using the powder samples demonstrate that both the hollow structure and the Au nanoparticles improve the water evaporation rates. Notably, the initial water evaporation rate of FCS@Au is higher than that of FCC (Fig. S13), but the trend reverses after the evaporation rate stabilizes, with FCC surpassing FCS@Au. This is attributed to the rapid temperature rise enabled by the Au-induced solar-thermal enhancement in the early stage (Fig. S10), followed by the continuous benefit of the nanoscale confinement in the hollow nanocage structure, facilitating the solar-thermal water evaporation. Ultimately, FCC reaches a stabilized rate of 2.11 kg m^−2^ h^−1^. Therefore, the hollow nanocage structure and the Au nanoparticles collaboratively enable FCC@Au to achieve a remarkable water evaporation rate of 2.56 kg m^−2^ h^−1^, significantly outperforming the control samples and among the reported highest values for 2D planar solar-thermal water evaporators [4, 7]. The evaporation coefficients (α) of FCC, and FCC@Au are calculated based on the HKS equation from the variations in mass and temperature recorded during the water evaporation process, with data collected in every 30 s over a total duration of 3000 s. The detailed calculation process can be found in the Text S1, and the calculation results are shown in Fig. S14. The statistical results show that the average evaporation coefficient of FCC@Au is 2.74 × 10^−4^, which is distinctly higher than 2.42 × 10^−4^ of FCC (Fig. 3c). This clearly indicates that the incorporation of Au nanoparticles enhances the water evaporation coefficient and thereby kinetically promotes the solar-thermal water evaporation process.

The multiphysics simulations using ANSYS Fluent are adopted to further verify that the enhanced evaporation performance originates from the physically induced directional water transport amplified by the nanoscale confinement and the Au nanoparticles. The simulation evaluates the flow, temperature, and pressure fields of a carbon nanocage model during the evaporation process (Text S2, Fig. S15). As shown in Fig. 3e, whether FCC@Au is immersed in water or in the vapor–liquid region, the heat generated by the Au nanoparticles achieves both temperature and pressure physical fields from the interior to the exterior of the mesoporous carbon shells, thus promoting the diffusion of water vapor through the nanopores and forming an orientation acceleration zone around the nanopores compared to the Au-free system (Figs. S16 and S17). Of greater interest is that, along with the evaporation and escape of water vapor through the nanopores of carbon shell above the water level, water spontaneously infiltrates the nanocage via the nanopores below the water level under the driving force of hydrostatic equilibrium and the principle of communicating vessels, which in turn reactivates water to generate new intermediate water for enhancing evaporation. This indicates the establishment of a directional evaporation process in which water is continuously activated and infiltrates the nanocage via the nanopores below the water level, followed by accelerated evaporation out of the nanocage through the nanopores above the water level under the action of physical fields, significantly benefiting the solar steam generation of FCC@Au.

When passing through the nanopores, water molecules experience altered intermolecular interactions due to the nano-confinement, which is elucidated by molecular dynamics (MD) simulation of bulk water and the water in confined nanopore (Fig. 3f), and the average numbers of hydrogen bonds per water molecule are calculated (Fig. 3g, h). Different form the nearly unchanged hydrogen bond number and water density across the bulk water, the hydrogen bond number decreases radially from 1.7 to 1 within the nanopore, indicating that, as water molecules approach the pore wall, their hydrogen bonding network experiences stronger confinement-induced disruption. Radial distribution functions further reveal a reduced water density near the pore walls, implying the disruption of the hydrogen bond network. These findings support the model illustrated in Fig. 1, where water transitions from bound water to intermediate water and finally to free water along the vertical direction from the solid–liquid interface. The weakened hydrogen bonding in the intermediate water layer facilitates its escape from the bulk, thereby enhancing the water evaporation in the confined system (Fig. 3i).

The water states inside the carbon nanocages are evaluated using the Raman spectra (Fig. S18). The Raman spectra can distinguish free water (FW) and intermediate water (IW). The presence of FCS in water can increase the IW/FW ratio from 0.332 to 0.521. After adding FCC that has more oxygen-containing groups and is more hydrophilic, the IW/FW ratio rises to 0.645, indicating that the increased hydrophilicity and the hollow structure make water more likely to evaporate. It is interesting that, in the FCC@Au/water system, the Au nanoparticles play a significant role in promoting water transport between the inner and outer regions as the laser irradiation induces local heating and an accompanying pressure rise inside the nanocages. The nano-confined effect promoted by the internal physical field perturbation of FCC@Au increases the IW/FW value further to 1.117. Meanwhile, FCC@Au exhibits a much lower water vaporization enthalpy of 1105 kJ kg^−1^ than those of both FCS and pure water (Fig. 3d), confirming the superior energy efficiency of the former for water evaporation.

To clarify the respective roles of nano-confinement and plasmonic effects, it is important to distinguish between intrinsic thermodynamic regulation and local field modulation. The reduction of the equivalent vaporization enthalpy and the enhanced evaporation kinetics primarily originate from the nano-confinement effect of the hollow carbon nanocages, which regulates the hydrogen bonding network and lowers the effective energy barrier for phase transition. The introduction of Au nanoparticles does not significantly increase the macroscopic photothermal heating of the system. Surface temperature measurements under identical 1 sun irradiation show nearly identical equilibrium temperatures for Au-containing and Au-free samples (Fig. S19), indicating that the overall heat generation is dominated by the broadband absorbing Fe–N–C carbon framework rather than the plasmonic component. Instead, Au nanoparticles mainly induce localized electromagnetic and thermal field fluctuations within the confined nanochannels. These localized physical fields enhance the interaction between interfacial water molecules and the carbon matrix, facilitating molecular activation and transport through the nano-confinement environment. As a result, the plasmonic component amplifies the effectiveness of nano-confinement rather than serving as an additional bulk heat source. Consistent with this interpretation, equivalent evaporation enthalpy measurements confirm that nano-confinement alone significantly reduces the effective phase transition barrier, while theoretical simulations reveal that localized field modulation further accelerates molecular escape dynamics. These complementary results establish a hierarchical promotion mechanism in which nano-confinement provides dominant thermodynamic regulation and plasmonic effects serve as a secondary amplification factor.

It should be noted that the primary objective of this work is not to maximize the apparent solar-to-vapor conversion efficiency through structural or optical optimization, but to elucidate the intrinsic regulation of interfacial water under nano-confinement and localized physical fields. In confined systems, the effective evaporation enthalpy can deviate from the bulk value due to hydrogen bond restructuring and molecular activation. Under such circumstances, efficiency values calculated using the standard bulk enthalpy may not fully reflect the actual thermodynamic and kinetic promotion of water evaporation. Therefore, in addition to reporting evaporation rates, we systematically evaluated the equivalent evaporation enthalpy and quantified the evaporation kinetics using the Hertz–Knudsen model to determine the evaporation coefficient. These analyses directly reveal the reduced energy barrier and accelerated molecular escape dynamics in the designed system.

### Catalytic Degradation Performances and Mechanisms of FCC and FCC@Au for BPA

**Fig. 4:**
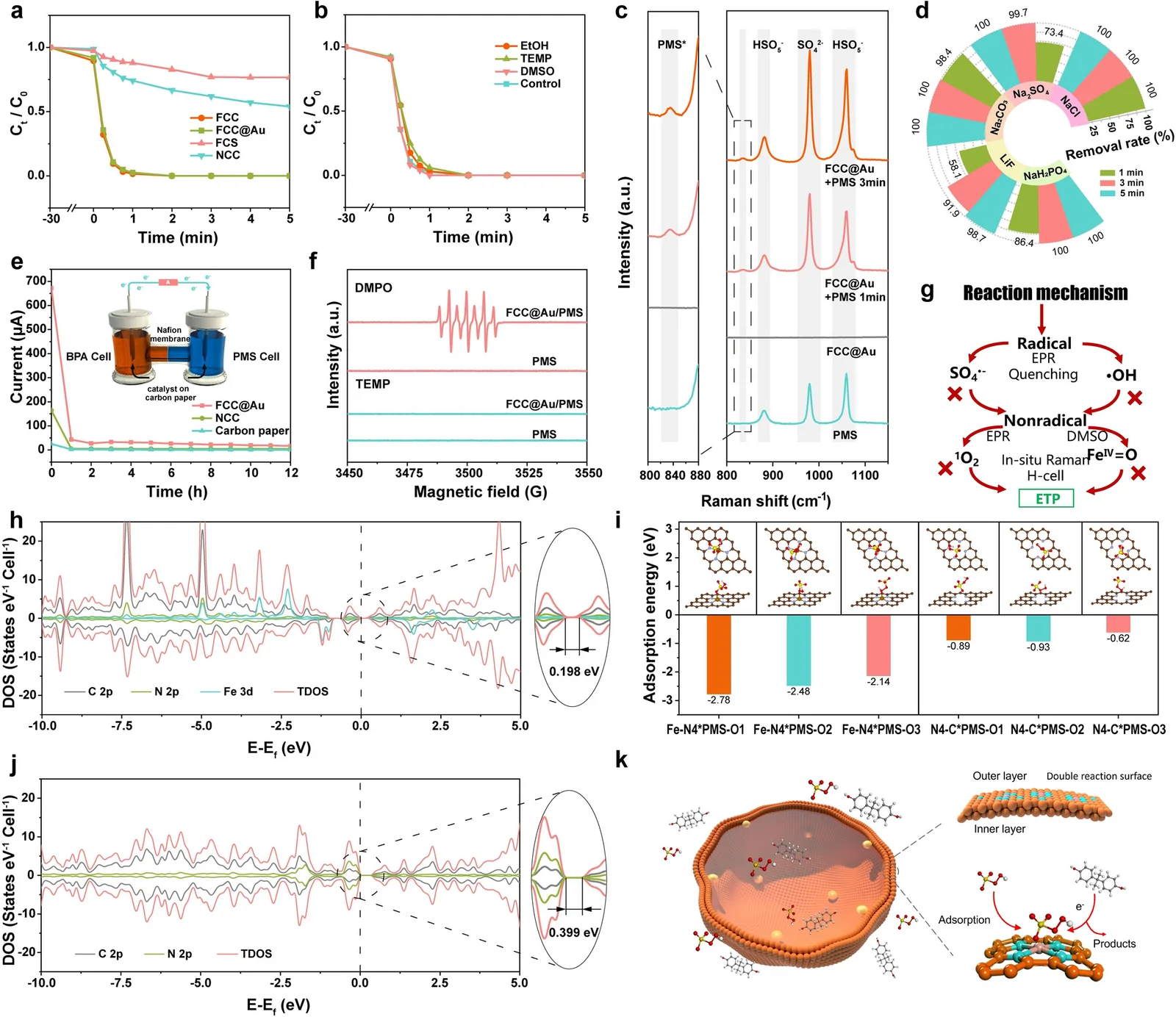
**a** Degradation efficiency of BPA by different catalysts (10 ppm BPA, 1 mg catalyst, 50 mL solution). **b** Radical quenching tests by using EtOH, DMSO, and TEMP. **c** In situ Raman spectra indicating the formation of PMS* intermediate on FCC surface. **d** Degradation efficiency of BPA under different ionic environments (10 mM). **e** Schematic and current responses of a dual-chamber electrochemical oxidation cell. **f** EPR spectra by using the trapping agents of DMPO and TEMP. **g** Exploration methods of the catalytic mechanism. **h** DOS of the Fe-N_4_-C. **i** Configurations and adsorption energies of different oxygen types of PMS adsorbed by different structures. **j** DOS of the N_4_-C. **k** Schematic diagram of the catalytic mechanism of FCC@Au

Bisphenol A (BPA) is a high-production-volume chemical, which is extensively utilized in producing polycarbonate plastics and epoxy resins. However, its persistent release into aquatic environments has led to growing ecological and health concerns, particularly due to its potent endocrine disrupting effects, which are detrimental to infants and children. Here, BPA is chosen as a representative model pollutant to evaluate the catalytic degradation performances of FCC and FCC@Au by activating PMS under ambient conditions. Figure 4a compares the degradation efficiencies of BPA by various catalysts in the presence of PMS. Among them, the FCC catalyst exhibits an exceptional performance, achieving complete degradation of 50 mL of 10 ppm BPA solution in less than 2 min with a catalyst dosage of as low as 1 mg, corresponding to an apparent rate constant (k_obs_) of 4.12 min^−1^, indicating that FCC is highly efficient in catalytic activation of PMS (Fig. S20). In the case of the FCC frame encapsulated with gold nanoparticles (denoted as FCC@Au), the complete BPA degradation within 2 min is still achieved.

The effect of PMS dosage on the catalytic degradation performance is shown in Fig. S21, where the degradation efficiency increases with increasing the PMS dosage until achieving a kobs of 3.65 min^−1^ with a PMS dosage of 0.4 g L^−1^. Assuming that all Fe atoms are active sites, the TOF calculation result is 2.2 × 10^4^ s^−1^. Note that, due to the difficulty in accurately quantifying the number of accessible Fe–N_4_ active sites, the TOF is estimated based on the total Fe content and should be considered as an upper-limit approximation. A comparative analysis with other PMS-based advanced oxidation processes (AOPs) reveals that the catalytic degradation efficiencies of FCC@Au significantly outperform most reported systems (Table S1). To gain mechanistic insights into the catalytic degradation pathway, radical quenching experiments are conducted by using specific scavengers: ethanol for hydroxyl and sulfate radicals, TEMP for singlet oxygen, and DMSO for high-valent metal species. As shown in Fig. 4b, none of the quenchers significantly inhibits the degradation activity, indicating that the typical reactive oxygen species (ROS) are not the primary active species in this system. This observation strongly suggests that the catalytic pathway is dominated by a non-radical mechanism, most likely an electron transfer process (ETP) between BPA and PMS mediated by the Fe–N_4_–C active sites within the FCC@Au framework.

To further validate the involvement of the ETP pathway, in situ Raman spectroscopy is used to monitor the interaction between PMS and the FCC@Au catalyst (Fig. 4c). Pure aqueous PMS solution exhibits distinct vibrational bands at 880 and 1060 cm^−1^, corresponding to the S–O stretching modes of HSO₅⁻, and at 980 cm^−1^ for SO_4_^2−^. In contrast, pristine FCC@Au has no discernible peaks in the range of 800–1150 cm^−1^, indicating no intrinsic Raman-active species in that range. Interestingly, upon introducing PMS into the FCC@Au system, a new band appears at 835 cm^−1^, which can be assigned to the formation of an activated PMS intermediate (PMS*) complex associated with electron transfer events. This spectral evolution provides direct spectroscopic evidence supporting the presence of PMS* species as transient electron acceptors on the catalyst surface.

To substantiate this mechanism further, a custom-designed electrochemical oxidation cell is constructed (Fig. 4e), comprising two chambers separated by a Nafion proton exchange membrane. Each chamber is equipped with a carbon paper electrode loaded with either FCC@Au or control material. One chamber contains the BPA solution while the other has PMS. The two electrodes are externally connected via a conductive wire linked to an ammeter to monitor current flow. Notably, a significant current signal (~ 168 μA cm^−2^) is observed in the FCC@Au system, whereas, the control experiments with either bare carbon paper or NCC (a catalyst lacking Fe–N_4_ sites) have negligible current signals of ~ 41 and ~ 6.25 μA cm^−2^, respectively. Over a reaction period of 12 h, 68% of the BPA is degraded in the FCC@Au system (Fig. S22), despite the physical separation from PMS. This electron-only communication across the external circuit provides compelling experimental evidence that BPA is oxidized via an ETP pathway, where PMS acts as the terminal electron acceptor. Additional mechanistic evidence is obtained via electron paramagnetic resonance (EPR) spectra (Fig. 4f). In the presence of the spin-trap DMPO, a characteristic seven line signal is observed, indicating the formation of PMS-derived radical-like species upon electron capture. When TEMP is used as a probe, however, no signal is observed, implying that singlet oxygen is not involved in the reaction, further corroborating the non-radical nature of the catalytic mechanism. Subsequently, the catalyst is subjected to open-circuit voltage tests. During the testing process, PMS and BPA are added successively. The results show that after adding PMS, the potential of FCC@Au increases to 0.95 V, while that of the NCC without iron increases to 0.78 V only, and the blank control group reaches only 0.24 V (Fig. S23). These results indicate that the doping of iron promotes the formation of PMS*, and the increase in potential suggests the transfer of charges to PMS*. After adding BPA, the potentials of both FCC@Au and NCC decrease, while the blank control group has no significant change. Taken together, the converging evidence from radical exclusion, in situ spectroscopy, and electrochemical validation establishes a surface-confined non-radical electron transfer pathway as the dominant mechanism in this system (Fig. 4c, g).

The DOS results further clarify the electronic advantages introduced by Fe–N_4_ sites. As shown in Fig. 4h, j, Fe incorporation significantly enriches the occupied states in the valence-band region (below − 2 eV), arising mainly from the hybridized Fe 3*d*–N 2*p* orbitals. The increased valence-state density enhances the ability of the Fe center to accept electrons from electron-rich pollutants, thereby facilitating the initial pollutant → Fe–N_4_ charge transfer step. Meanwhile, the Fe–N_4_ catalyst maintains only a moderate DOS around the Fermi level. Notably, the band gap near E_f_ decreases from 0.399 eV (Fe-free system) to 0.198 eV upon Fe coordination, indicating a more accessible and better-aligned electron transfer channel. Moderately localized Fe-centered d states serve as discrete electron relays that promote stepwise pollutant → Fe–N_4_ → PMS electron transfer, while the regulated DOS near the Fermi level prevents excessive electron injection that would otherwise induce O–O homolysis toward radical pathways. The regulated DOS at the Fermi level enables selective PMS reduction, while the enriched occupied states below Ef facilitate efficient electron capture from pollutants; together, these features establish Fe–N_4_ as an effective electron mediator.

Further adsorption energy calculations in Fig. 4i reveal that Fe–N_4_–C possesses a substantially stronger affinity toward PMS than N_4_–C, with the most stable O-binding configuration reaching an adsorption energy as low as − 2.78 eV. In contrast, N_4_–C fails to form a stable Fe–O–PMS coordination, explaining its inability to initiate subsequent pollutant degradation. The simulated charge distribution maps further distinguish the electronic features of Fe–N_4_ from N_4_–C. In Fe–N_4_, the metal center generates a pronounced four-lobed charge accumulation aligned with the Fe–N coordination directions, reflecting the participation of Fe 3*d* orbitals and the strong electronic coupling between Fe and the surrounding N atoms. This *d–p* hybridization produces an electronically polarized microenvironment that facilitates PMS adsorption through both electrostatic attraction and Fe–O coordination. In contrast, N_4_–C shows only weak and isotropic charge localization around the pyridinic N atoms, without the directional electronic lobes observed in Fe–N_4_. The absence of metal-centered d orbitals prevents the formation of strong PMS–surface interactions, consistent with the much weaker adsorption energy and the inability of N_4_–C to polarize the O–O bond of PMS (Fig. S24). Upon the adsorption of PMS, Fe–N_4_ undergoes a remarkable electronic reconstruction, as evidenced by the DOS results (Figs. S25 and S26). The Fe 3*d* orbitals, particularly d_z_^2^/d_xz_ and d_yz_, exhibit pronounced enhancement near the Fermi level, forming hybridized Fe–O(PMS) states. These new coordination states narrow the gap between occupied and unoccupied 3*d* levels and generate energetically accessible half-filled orbitals that mediate electron acceptance and donation. Simultaneously, the PMS O-2*p* orbitals shift upward toward the Fermi level and strongly overlap with Fe-3*d* within − 1 to + 1 eV, enabling direct Fe → PMS electron transfer while avoiding O–O homolytic cleavage.

Work-function and frontier-orbital analyses corroborate this electron transfer pathway (Figs. S27 and S28). The HOMO of BPA (− 5.25 eV vs. vacuum) lies higher than the Fermi level of PMS-adsorbed Fe–N_4_ (work function: − 6.339 eV), making the electron donation from BPA to Fe–N_4_*PMS thermodynamically feasible. In contrast, pristine Fe–N_4_ exhibits a much higher Fermi level (− 4.028 eV), insufficient to accept electrons from BPA. These results collectively confirm the directionality of the non-radical ETP: BPA → Fe–N_4_ → PMS, driven by PMS-induced modulation of the Fe–N_4_ electronic structure.

The proposed ETP-based mechanism is schematically illustrated in Fig. 4k. Both PMS and BPA are adsorbed onto the surface of the FCC@Au catalyst. Electrons are transferred from BPA to the Fe–N_4_ active sites and subsequently to the adsorbed PMS molecules, forming the PMS* intermediate that drives the oxidative degradation of BPA. One of the most significant advantages of this non-radical mechanism is its strong environmental adaptability. As demonstrated in Figs. 4d and S29, the FCC@Au catalyst retains its high catalytic performance across a broad range of pH conditions (from acidic to alkaline) and in the presence of various inorganic ions commonly found in environmental water. To further evaluate the environmental adaptability of the system, a mixed-pollutant solution containing RhB, OFL, IBU, BPA, and CBZ (10 ppm each) is employed. The FCC@Au catalyst exhibits efficient degradation toward all pollutants, demonstrating strong tolerance to complex contaminant environments (Fig. S30). The excellent performance stability under diverse conditions underscores the practical applicability of the catalyst in real-world water treatment scenarios.

### Constructing 3D Multifunctional FCC@Au-based Evaporator

**Fig. 5:**
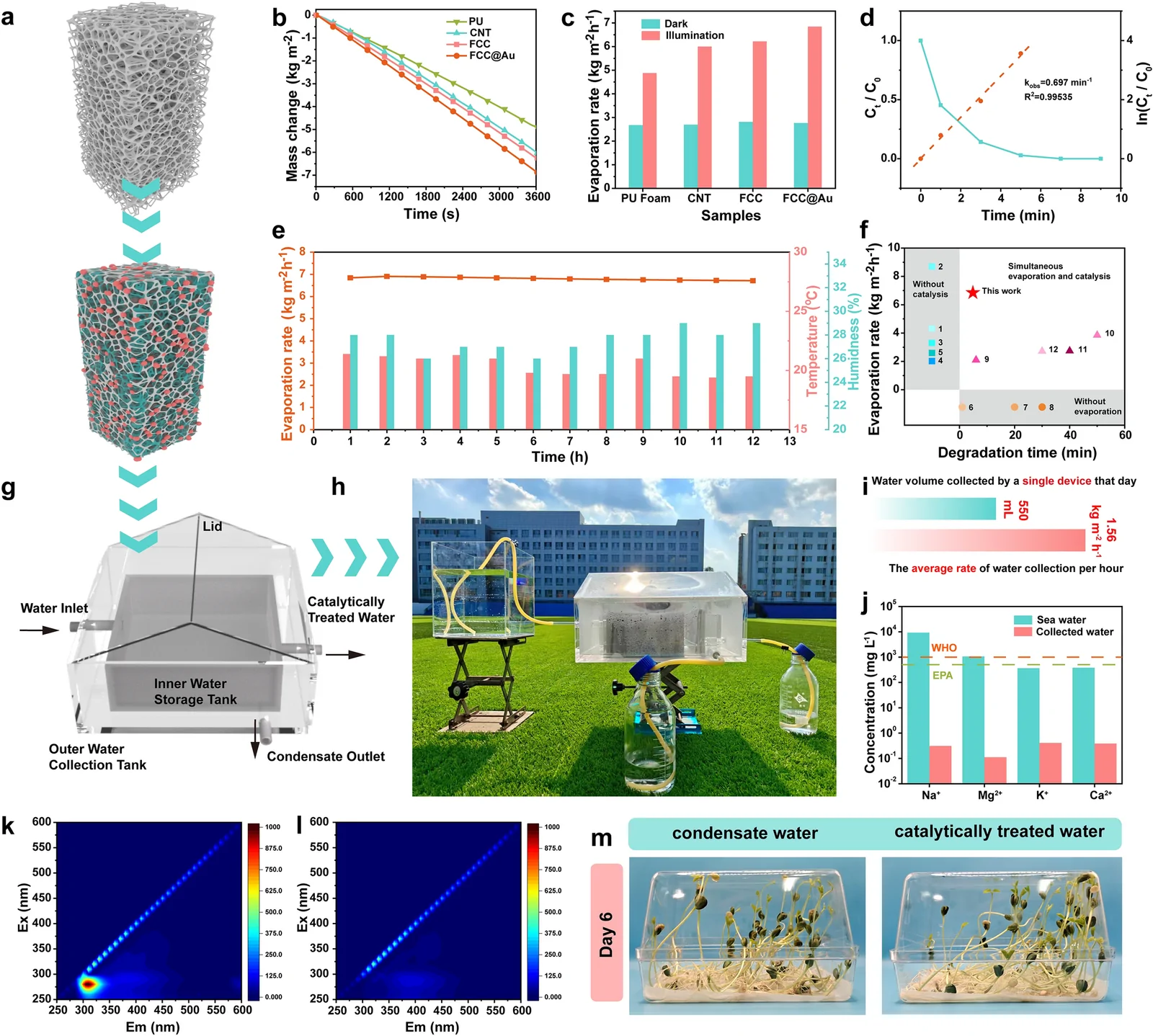
**a** Schematic illustrating the FCC@Au-coated PU foam. **b** Mass change curves of PU, CNT, FCC, and FCC@Au under 1-sun irradiation. **c** Comparison of water evaporation rates in dark and under solar light irradiation. **d** Degradation kinetics of BPA catalyzed by FCC@Au- coated PU foam. **e** Long-term cycling test of FCC@Au-loaded PU foam under 1-sun irradiation. **f** Comparison of catalytic degradation and solar evaporation properties of FCC@Au-loaded PU foam with those of counterparts reported. **g** Schematic diagram of the collection device model. **h** Photograph of outdoor solar evaporation setup under natural sunlight. **i** Total amount of fresh water collected on the test day and the corresponding rate. **j** Comparison of ion concentrations in seawater (Yellow Sea) and condensate water. 3D fluorescence spectra of seawater containing BPA **k** before and** l** after catalytic degradation treatment. **m** Seed germination tests using condensate water and catalytically treated water over 6 days

To demonstrate the practical applicability of FCC@Au, a multifunctional 3D evaporator is designed and fabricated to simultaneously enhance solar-driven water evaporation and catalytic degradation of organic pollutants (Figs. 5a and S31). Commercially available polyurethane (PU) foam (Fig. S32) is selected as the structural scaffold because of its superabsorbent and lightweight features. Briefly, FCC@Au powders are first ultrasonically dispersed in water, and then deposited onto the PU foam uniformly via a trickle impregnating method. To ensure strong adhesion and stability, the FCC@Au coated foam is sequentially sprayed with an aqueous polyvinyl alcohol (PVA) solution and a glutaraldehyde solution (adjusted to pH = 2) to induce crosslinking. The SEM image shows that The PU foam is uniformly coated with an FCC@Au layer on its surface (Fig. S33). Owing to the unique structure of the hollow carbon nanocage embedded with gold nanoparticles, the FCC@Au-loaded PU foam evaporator exhibits an exceptional solar evaporation rate of 6.84 kg m^−2^ h^−1^ under 1-sun irradiation, significantly outperforming both the FCC-loaded PU foam (6.22 kg m^−2^ h^−1^) and a benchmark carbon-based solar-thermal material—CNTs (5.99 kg m^−2^ h^−1^) under the same conditions (Fig. 5b). Meanwhile, the FCC@Au sample has an evaporation rate of 6.50 kg m^−2^ h^−1^when evaporating seawater (from the Yellow Sea, China) (Fig. S34). The superior performance of FCC@Au is attributed to the enhanced broadband light absorption enabled by the synergistic effect of plasmonic Au nanoparticles and the hollow carbon structure, the high thermodynamic activity of water ascribed to the nano-confined effect of the mesoporous carbon shell, and the high evaporation kinetic activity attributed to localized physical field modulation from the interior of the nanocage to the exterior of its shell induced by the solar irradiation of the Au nanoparticles embedded on the inner surface of the nanocage, which reduces the vaporization enthalpy of water molecules and promotes the water evaporation coefficient.

Dark evaporation experiments further confirm the reduction in water vaporization enthalpy (Fig. 5c). Compared to PU foam and CNT/PU samples, the PU foams loaded with FCC and FCC@Au exhibit slightly higher dark evaporation rates, indicating a thermodynamically favorable water evaporation process. Upon solar light irradiation, FCC@Au exhibits a significant increase in water evaporation rate, which is ascribed to the local physical field-enhanced nano-confinement within the carbon nanocage. Furthermore, the excellent hydrophilicity of the 3D foam (Fig. S35) ensures continuous water supply to the evaporation interface. As a result, the FCC@Au-loaded PU foam is still efficient in solar steam generation under higher solar light intensities, achieving a water evaporation rate of 16.86 kg m^−2^ h^−1^ under 4-sun irradiation (Fig. S36). During a 12 h continuous solar evaporation under ambient conditions (∼20 °C, relative humidity ∼27%), the foam evaporator exhibits an outstanding stability, and the evaporation rate maintains no less than 6.8 kg m^−2^ h^−1^ (Figs. 5e and S37). The planar (2D) configuration is used to evaluate the intrinsic evaporation capability of the material, where the performance enhancement is governed by nano-confinement-induced thermodynamic and kinetic regulation of interfacial water. In contrast, the higher evaporation rate observed in the 3D architecture primarily originates from the geometric effects, including the increased liquid–vapor interfacial area and improved mass transport. Importantly, when different photothermal materials are integrated into the same 3D substrate, FCC@Au consistently exhibits superior performances compared to PU, CNT, and FCC, confirming that the intrinsic material design plays the dominant role, while the 3D architecture serves as an amplification platform.

In addition to the efficient and stable water evaporation, the pollutant degradation capacity of the FCC@Au-loaded PU foam is essential for its applications in purifying wastewater. In a simulated wastewater purification test with PMS as the oxidant, the foam evaporator is partially immersed (∼3 cm in depth) in 50 mL BPA solution (10 ppm). As shown in Fig. 5d, complete degradation of BPA is achieved within 7 min with an apparent rate constant of 0.697 min⁻^1^. Notably, the catalyst maintains stable degradation performance over five consecutive cycles, indicating excellent recyclability (Fig. S38). The comparative performance analyses (Fig. 5f, Table S3) reveal that the FCC@Au-loaded foam evaporator outperforms most reported catalysts dedicated solely to pollutant degradation, and simultaneously has better solar steam generation efficiency than conventional solar-thermal evaporators [3, 4, 8–10, 31–37]. Notably, even compared to existing dual-function materials designed for both solar evaporation and pollutant removal, FCC@Au exhibits significantly superior integrated performances, establishing itself as a leading candidate for multifunctional solar-driven water purification application.

### Practical Application in Simulated Real-World Scenarios

To simulate real-world application scenarios, a customed outdoor experimental setup is constructed for water purification as illustrated in the schematic diagram (Figs. 5g and S39), mainly consisting of a lid, tubing, an inner chamber, and an outer chamber. The inner chamber is used to hold seawater or wastewater, and connected to the external environment via two tubes positioned at the top and bottom, enabling water supply and collection of treated effluent after catalytic degradation of pollutants; whereas, the outer chamber and the lid are designed for condensing water vapor and collecting the clean distilled water. A tube located at the lower end of the outer chamber allows for the extraction of the collected clean water. A photograph of the water purification device in operation is shown in Fig. 5h. The outdoor water purification test was conducted on June 5, 2025 in Chaoyang District, Beijing under the weather conditions detailed in Fig. S40. The evaporator has an effective area of 440 cm^2^, consisting of 22 pieces of the PU foam loaded with FCC@Au (100 × 100 × 20 mm^3^). Each piece is inserted into the inner chamber with 4 cm extending above the water surface. During the 8h test from 8:00 a.m. to 4:00 p.m., a total of 550 mL of evaporated water was collected. Based on the collected volume and the exposure area, the average water collection rate is calculated to be 1.56 kg m^−2^ h^−1^ (Fig. 5i). It should be clarified that the laboratory and outdoor tests are conducted under fundamentally different configurations. The laboratory evaluation is performed in an open system under a calibrated 1 sun solar simulator, where the generated vapor freely diffuses into ambient air. Under such conditions, the measured evaporation rate primarily reflects the intrinsic photothermal conversion and interfacial water regulation capability of the evaporator. In contrast, the outdoor test is carried out in a closed water collection device, in which evaporation and condensation occur simultaneously within a confined space. In this configuration, vapor accumulation increases the local partial pressure, thereby reducing the vapor pressure gradient that drives evaporation. Consequently, the practical water collection rate is jointly governed by evaporation, vapor transport, and condensation efficiency rather than solely by the intrinsic evaporation performance of the material. Therefore, the laboratory measurements are intended to evaluate the intrinsic evaporation capability of the FCC@Au/PU evaporator, whereas the outdoor experiment serves as a practical demonstration of simultaneous desalination and pollutant removal under realistic conditions rather than a fully optimized device-level water production system. These results confirm the practical potential of the solar-thermal FCC@Au evaporator. The quality of the collected water is analyzed by comparing the concentrations of four representative cations in the raw Yellow Sea water (China) with those of the collected clean water. Clearly, the purification process achieves high efficiencies of exceeding 99.85% in removing the four ions, meeting the World Health Organization (WHO) standards for drinking water (Fig. 5j).

Subsequently, to evaluate the catalytic degradation capability under real seawater conditions, a bisphenol A (BPA) solution (10 ppm) is prepared with seawater from the Yellow Sea as the solvent. After the catalytic degradation for 15 min, the 3D fluorescence spectra show that the characteristic BPA signal in the lower-left region disappears (Fig. 5k, l), while the signal attributed to humic substances naturally present in seawater remains largely unchanged, indicating that the catalytic degradation system exhibits strong resistance to environmental interferences and preferentially targets pollutants like BPA with electron-donating characteristics.

To assess the biological safety of different water sources, soybean seed germination tests with 45 seeds in each group are carried out by using condensate water, seawater, catalytically treated water, and BPA solution. As shown in Fig. S41, the germination rate of the seeds irrigated with condensate water increases rapidly to 97.7% within 4 days and remains stable thereafter, accompanied by vigorous root and shoot growth. A similar germination trend is observed for seeds watered with the catalytically treated water (with a germination rate of 95.5%), confirming that the purified water is free of phytotoxic effects. In stark contrast, no germination occurs in the seawater group throughout the 6-day period due to the osmotic inhibition. The seeds exposed to the BPA solution exhibit severely suppressed germination, with less seeds sprouting, and the seedlings show stunted growth and morphological abnormalities. The photographic records (Figs. 5m and S42) provide direct visual evidence that the FCC@Au system delivers clean water with sufficient quality to sustain healthy growth of plants. Overall, the evaporator demonstrates dual functionality: it can degrade organic pollutants in seawater or wastewater via an advanced oxidation process to produce safely dischargeable pretreated water, and it can also generate high-purity clean water for human consumption through solar-thermal evaporation and condensation. These features highlight its promising potential for practical environmental applications.

## Conclusion

This study addresses a long-standing bottleneck in solar-driven interfacial evaporation by first time creating a multifunctional solar-thermal material that integrates efficient nano-confined solar-thermal evaporation boosted by physical field disturbance coupled with non-radical advanced oxidation pollutant remediation process. The rationally designed functionalized solar-thermal material consists of hollow mesoporous carbon nanocages embedding Au nanoparticles in the inner wall and doped with atomically dispersed Fe–N_4_ sites (FCC@Au). The nanoscale confinement within the mesoporous channels promotes the generation of intermediate water induced by the amplified interface effect from the solid–liquid pore interface, while the localized surface plasmon resonance from the encapsulated Au nanoparticles perturbs directional thermal and mass transport fields from the inside out to enhance the diffusion and evaporation kinetic activities of intermediate water, collectively lowering the energetic barrier and enhancing kinetics evaporation coefficient for water evaporation. Furthermore, more water can spontaneously infiltrate the nanocage via the nanopores under the driving force of hydrostatic equilibrium and the principle of communicating vessels, therefore, forming the evaporation process that water is continuously activated in the process of passing through the nanopores followed by accelerated evaporation out of the sphere boosted by physical field disturbance. As a result, the FCC@Au-loaded PU foam solar-thermal evaporator achieves high evaporation rates of 2.56 kg m^−2^ h^−1^ in a 2D planar evaporator and 6.84 kg m^−2^ h^−1^ in a 3D configuration under 1-sun irradiation. Beyond the evaporation, the Fe–N_4_ sites can catalytically activate PMS via a non-radical electron transfer pathway, enabling ultrafast and selective degradation of BPA pollutant with a mass-normalized rate constant of 182.5 L g^−1^ min^−1^, far surpassing state-of-the-art catalysts. FCC@Au is compatible with the fabrication of various 3D evaporation architectures and can be readily integrated into water collection systems for practical applications. This pioneering study systematically couples interfacial water structure regulation with nanoscale confinement to reduce water vaporization enthalpy, offering a new strategy for enhancing water evaporation performances. This work demonstrates the potential of integrating multifunctional capabilities into solar-thermal materials, paving the way for future innovations in material design for solar-driven water purification.

## Supplementary Information

Below is the link to the electronic supplementary material.

Supplementary file1 (DOCX 6424 KB)
