# Supplementary material for: Nano-Confined Solar-Thermal Water Purification Boosted by Physical Field Disturbance Coupled with Ultrafast Non-Radical Advanced Oxidation Process
Source: Nanomicro Lett. 2026 Jul 6;18:424. doi: 10.1007/s40820-026-02249-x (PMC13338077; doi:10.1007/s40820-026-02249-x)
Supplement: Supplementary file 1 — Supplementary file1 (DOCX 6424 KB) [file 40820_2026_2249_MOESM1_ESM.docx]

Supporting Information for

**Nano-Confined Solar-Thermal Water Purification Boosted by Physical-Field Disturbance Coupled with** **Ultrafast Non-Radical Advanced Oxidation Process**

Fan-Zhen Jiao^1,2^, Xiaoyang Fang^1^, Sheng-Xing Hou^1^, Zhi-Hao Wang^2^, Wenbo You^3^, Zhenzhong Yang^4*^, Zhong-Zhen Yu^2*^, Jin Qu^1*^

^1^ State Key Laboratory of Organic−Inorganic Composites, Beijing University of Chemical Technology, Beijing 100029, P. R. China

^2^ Center for Nanomaterials and Nanocomposites, College of Materials Science and Engineering, Beijing University of Chemical Technology, Beijing 100029, P. R. China

^3^ Shanghai Key Laboratory of Atmospheric Particle Pollution and Prevention, Department of Environmental Science and Engineering, Fudan University, Shanghai 200433, P. R. China

^4^ Department of Chemical Engineering, Tsinghua University, Beijing 100084, P. R. China

*Corresponding authors. E-mail: [yangzhenzhong@tsinghua.edu.cn](mailto:yangzhenzhong@tsinghua.edu.cn) (Zhenzhong Yang); [yuzz@mail.buct.edu.cn](mailto:yuzz@mail.buct.edu.cn) (Zhong-Zhen Yu); [qujin@buct.edu.cn](mailto:qujin@buct.edu.cn) (Jin Qu)

**Supplementary Text**

**Text S1 Calculation of Evaporation Coefficient Based on the Hertz–Knudsen–Schrage Equation**

The evaporation coefficient (*α*) was determined from the experimental measurements of mass changes and the surface temperature during the solar-thermal water evaporation process.

The instantaneous mass flux (*J*) during water evaporation was obtained from the slope of a recorded mass–time curve:

$J=-\frac{1}{A}\frac{dm}{dt}$ (S1)

where *A* is the effective evaporation area, and $\frac{dm}{dt}$is the mass change rate.

The saturated vapor pressure at the surface temperature *T* was calculated using the Antoine equation:

$\log_{10} p_{sat}(T)=A-\frac{B}{C+T}$ (S2)

The actual vapor pressure in the surrounding air was obtained by multiplying the saturated vapor pressure at the ambient temperature (27 °C) with the relative humidity (67%).

$p_{v}=RH\cdot p_{sat}(T_{amb})$ (S3)

The relationship between the mass flux and the evaporation coefficient is expressed by the Hertz–Knudsen–Schrage (HKS) equation [S1]:

$J=\frac{2\alpha}{2-\alpha}\sqrt{\frac{M}{2\pi RT_{i}}}\left( p_{\text{sat}}\left( T_{i} \right)-p_{v} \right)$ (S4)

where *M* is the molar mass of water, and *R* is the universal gas constant.

By substituting the experimentally determined *J*, the calculated $p_{\text{sat}}\left( T_{i} \right)$, and the ambient vapor pressure $p_{v}$into the equation above, the value of the evaporation coefficient *α* was obtained.

**Text S2 Details of Physical Field Simulation**

To investigate the internal flow dynamics and heat transfer behaviors of the solar-thermal evaporator, multiphysics simulations were performed using the Ansys Fluent software. The multiphase flow, radiation, and heat transfer modules were employed to simulate the velocity field, temperature field, and pressure field during the evaporation. A two-phase model consisting of liquid water and water vapor was constructed. The simulation was carried out using the finite volume method (FVM) to ensure high accuracy. The simulation geometry was set as a 400 nm × 400 nm domain, discretized into approximately 12,000 unstructured mesh elements using ANSYS Meshing (Fig. S15) with a temporal step size of 0.001 s.

To simulate the seawater evaporation process, Fluent 2021R1 was used to import the mesh, conFig. multiphase models, and apply boundary conditions. Due to the clearly distinguishable interface between liquid and vapor phases, the VOF (Volume of Fluid) model was selected to track the phase boundary. Flow behavior was assumed to be laminar. The governing equations for mass, momentum, and energy conservation are as follows [S2]:

Continuity Equation:

$\frac{\partial\rho}{\partial t}+\nabla\cdot(\rho\vec{U})=0$ (S5)

where *ρ* is the fluid density, $\vec{U}$ is the velocity vector, and *t* is time.

Momentum Equation:

$\frac{\partial(\rho u)}{\partial t}+\nabla\cdot(\rho u\vec{U})=\nabla^{2}(\mu_{\text{eff}}u)-\nabla p$ (S6)

where *μ*_eff_ is the effective viscosity, and *p* is the pressure.

Energy Equation:

$\frac{\partial(\rho h)}{\partial t}+\nabla\cdot(\rho h\vec{U})=\nabla^{2}(\lambda_{\text{eff}}T)$ (S7)

where *h* is the specific enthalpy, *λ*_eff_ is the effective thermal conductivity, and *T* is the local temperature.

Mass transfer between the liquid and vapor phases was modeled using the Lee model, which relates the phase change rate to the temperature difference between the local fluid and its saturation temperature. The evaporation and condensation mass transfer rates are given by [S3]:

Evaporation:

$\dot{m}_{\text{lv}}=coeff_{\text{from}}\cdot\alpha_{l}\rho_{l}\frac{T_{m}-T_{s}}{T_{s}}$ (S8)

Condensation:

$\dot{m}_{\text{vl}}=coeff_{\text{to}}\cdot\alpha_{v}\rho_{v}\frac{T_{s}-T_{m}}{T_{s}}$ (S9)

These coefficients determine the strength of phase transition. Larger values accelerate convergence toward thermal equilibrium and ensure that the local temperature approximates Ts during phase change.

The Surface-to-Surface (S2S) radiation model was applied to simulate radiative exchange in the enclosed domain. The model assumes no participating medium and calculates energy transfer between surfaces based on view factors. This model is particularly suitable for gray, diffuse surfaces and enclosed environments. Compared with DTRM and DO models, S2S is computationally efficient for steady-state simulations, especially when surface-to-surface radiation dominates and gas-phase absorption or scattering can be ignored. The radiative heat exchange depends on geometric relationships between surfaces, captured by the view factor, which quantifies energy transfer based on orientation, distance, and relative surface area.

**Text S3 Molecular Dynamics Simulation Details and Methods**

In the present work, the situation of water in confined and unconfined environments was studied using molecular dynamics simulations. System 1 is composed of CNTs containing 193 hydroxyl groups and 2673 water molecules. System 2 contains only 2673 water molecules. The simulations were performed using the Gromacs-2023.5 software package and the molecular force field was performed using GAFF2 [S4, S5]. The results were visualized using the visualization software VMD [S6].

The non-bonding interactions contain van der Waals and electrostatic interactions, both of them have a cut-off radius set to $r_{c}=1.2$ nm. The interaction between particles is expressed using the LJ potential.

$V_{\mathrm{LJ}}=\left\{ \begin{aligned} 4\epsilon[{(\frac{\sigma}{r})}^{12}-{(\frac{\sigma}{r})}^{6}], r<r_{c} \\ 0, r\geq r_{c} \end{aligned} \right.$ (S10)

where $\epsilon$ and $\sigma$ denote the strength of the inter-particle interaction and the particle size, respectively, depending on the force field chosen. The electrostatic interaction between particles is expressed as the Coulomb potential:

$V_{\mathrm{Coulomb}}=\left\{ \begin{aligned} \frac{1}{4\pi\varepsilon_{0}}\frac{q_{i}q_{j}}{\varepsilon_{r}r}, r<r_{c} \\ 0, r\geq r_{c} \end{aligned} \right.$ (S11)

where $\varepsilon_{0}$ and $\varepsilon_{r}$ denote the vacuum dielectric constant and relative dielectric constant, respectively, and *q* is the charge carried by the particle. Restrained Electrostatic Potential (RESP) method was used to calculate atomic charges [S7]. Long range electrostatic interactions were treated using the particle mesh Ewald (PME) method [S8].

First, energy minimization was performed using the steepest descent method with energies and forces of 0.01 kJ/mol and 1000 kJ/mol/nm. Next, MD was run for 125,000 steps of 1fs, which is a constant volume process using a Berendsen thermostat. MD integrates Newton's equations using the velocity-Verlet algorithm. Then, MD was run for 250,000 steps of 2 fs, using Berendsen for both the thermostat and pressure coupling. Finally, MD was run for 50,000,000 steps of 2 fs, using V-rescale and C-rescale for the thermostat and pressure coupling, respectively. The time constants for temperature coupling and pressure coupling are 1 ps and 2 ps, respectively. The simulations used periodic boundary conditions (PBC). The temperature of the simulated system was 298.15 K and the pressure was 1 atm.

**Text S4 DFT Calculations Details and Methods**

Density functional theory (DFT) calculations were conducted using the Vienna Ab initio Simulation Package (VASP) [S9]. The electron–ion interactions were treated with the projector augmented wave (PAW) method at a plane-wave cutoff energy of 450 eV [S10]. Exchange–correlation effects were described using the Perdew–Burke–Ernzerhof (PBE) functional within the generalized gradient approximation (GGA) [S11]. Geometry optimizations employed a force convergence criterion of 0.03 eV Å⁻¹ and an electronic energy threshold of 10⁻⁵ eV. To properly account for the localized d electrons of transition-metal centers, the GGA+U approach was applied [S12]. Brillouin-zone sampling was performed using a 4 × 3 × 1 Monkhorst–Pack k-point mesh. A vacuum layer of 15 Å along the z-direction was introduced to eliminate spurious interactions between periodic images. Long-range dispersion forces were incorporated through the DFT-D3 scheme with Becke–Johnson damping [S13].

**Text S5 Scalability and Cost Considerations**

From a practical perspective, the synthesis strategy employed in this work is based on solution-phase assembly and template-assisted pyrolysis, which are widely used in the scalable production of carbon-based functional materials. The key precursors, including iron salts, dopamine, silica templates, and polymeric reagents, are commercially available and relatively inexpensive. Moreover, the fabrication process does not require high-pressure systems, sophisticated lithographic techniques, or complex multistep purification procedures, which is favorable for batch production.

Although Au nanoparticles are incorporated as plasmonic components, their loading amount in the present system is extremely low (~0.13 wt% as determined by ICP analysis). Based on the current market price of gold (~60 USD g⁻¹), the Au contribution corresponds to only ~0.08 USD per gram of composite material. The remaining components, mainly consisting of carbon precursors and iron-based species, are low-cost and contribute approximately 1–2 USD per gram at laboratory scale. Therefore, the overall material cost is estimated to be within ~1–2 USD per gram. Considering that only ~20 mg of active material is required for a 3D evaporator unit (2 × 1 × 6 cm³), the material cost per device is well below 0.05 USD, excluding the inexpensive polyurethane substrate.

Importantly, the plasmonic component functions primarily as a localized physical-field modulator rather than the dominant photothermal absorber. Therefore, its loading can be further optimized, and alternative conductive or plasmonic nanostructures may be employed to reduce material cost without altering the catalytic mechanism. In addition, the synthesis protocol is compatible with scalable coating approaches (e.g., dip-coating or spray deposition), enabling integration into large-area evaporative modules. These considerations suggest that the proposed architecture is potentially scalable and economically viable for practical solar-driven water treatment applications.

**Supplementary Figures**


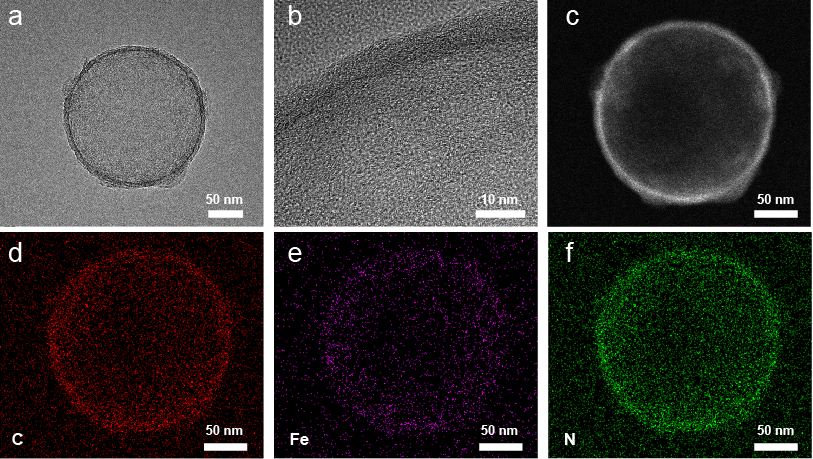


**Fig. S1** a–c) TEM and HAADF-STEM images of FCC nanoparticles at different magnifications; d–f) Corresponding elemental mapping results of C, Fe, and N elements, confirming the uniform distribution of constituent elements within the FCC nanostructure.


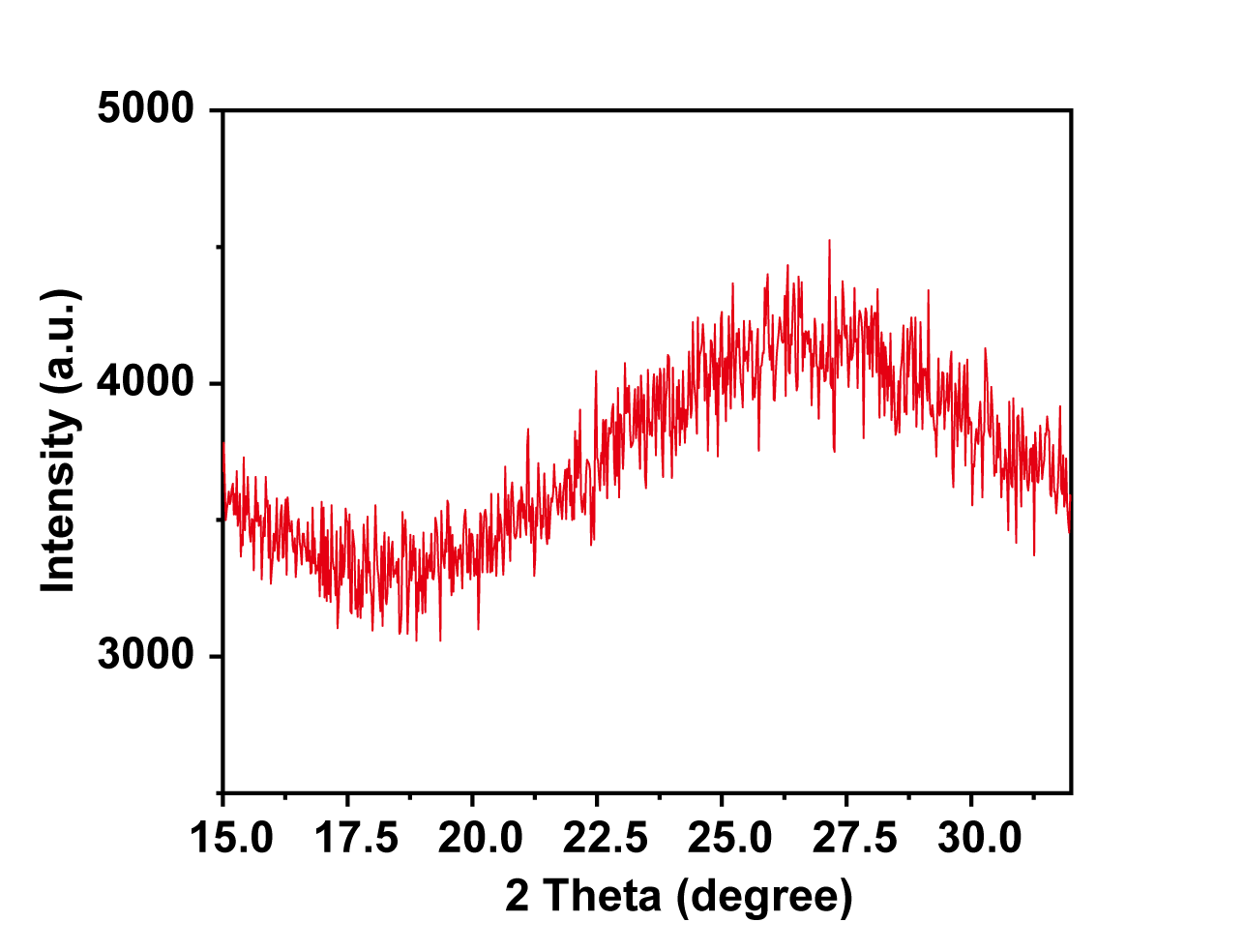


**Fig. S2** The magnified partial view of the XRD spectrum of FCC@Au.


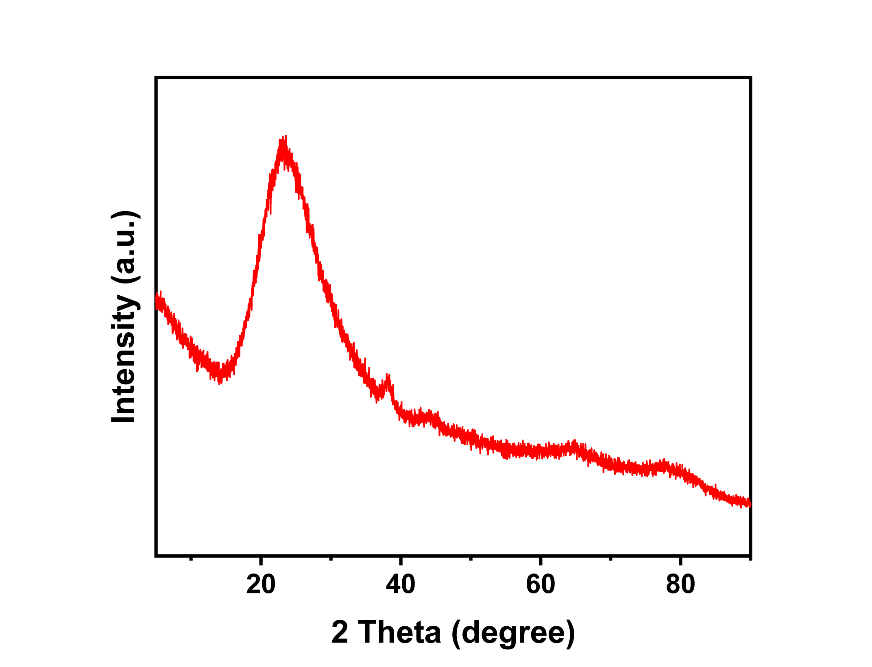


**Fig. S3** XRD spectrum of silica nanospheres.


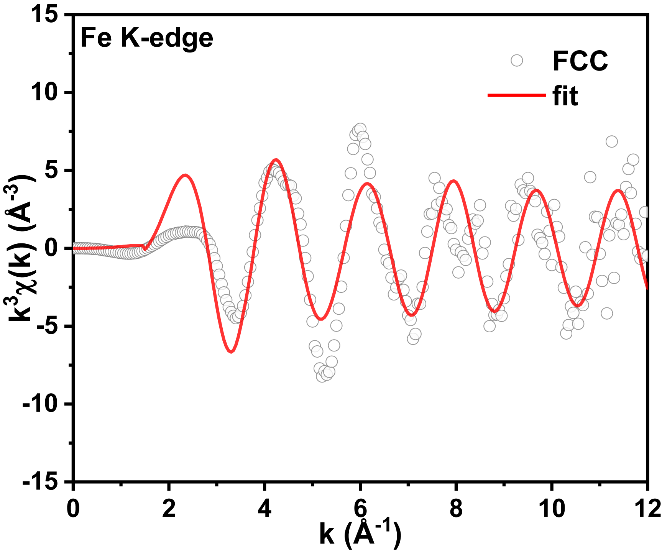


**Fig. S4** EXAFS spectrum of FCC at the Fe K-edge in k-space.


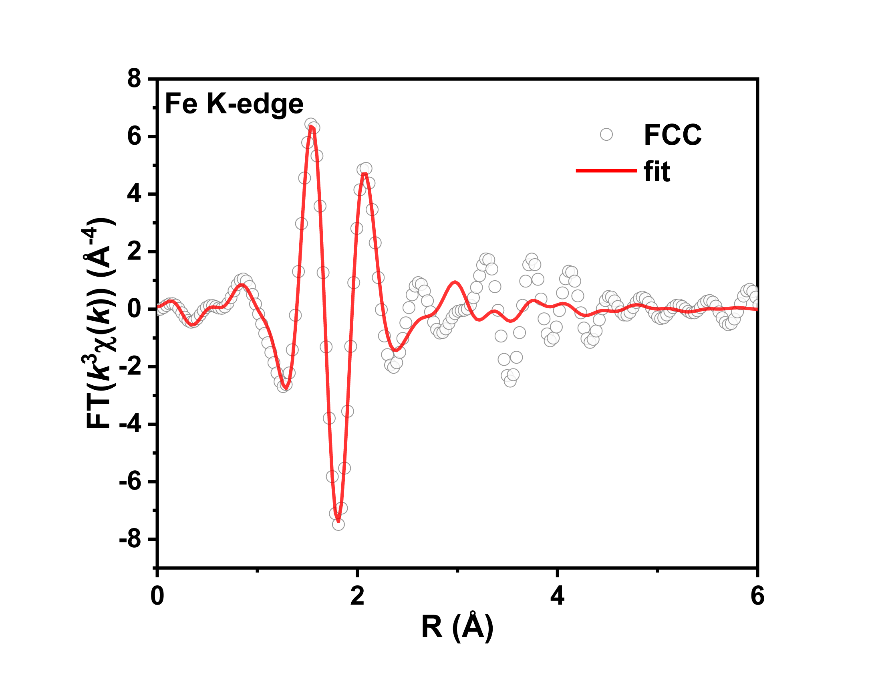


**Fig. S5** Re(k^3^χ(k)) oscillation curve of FCC.


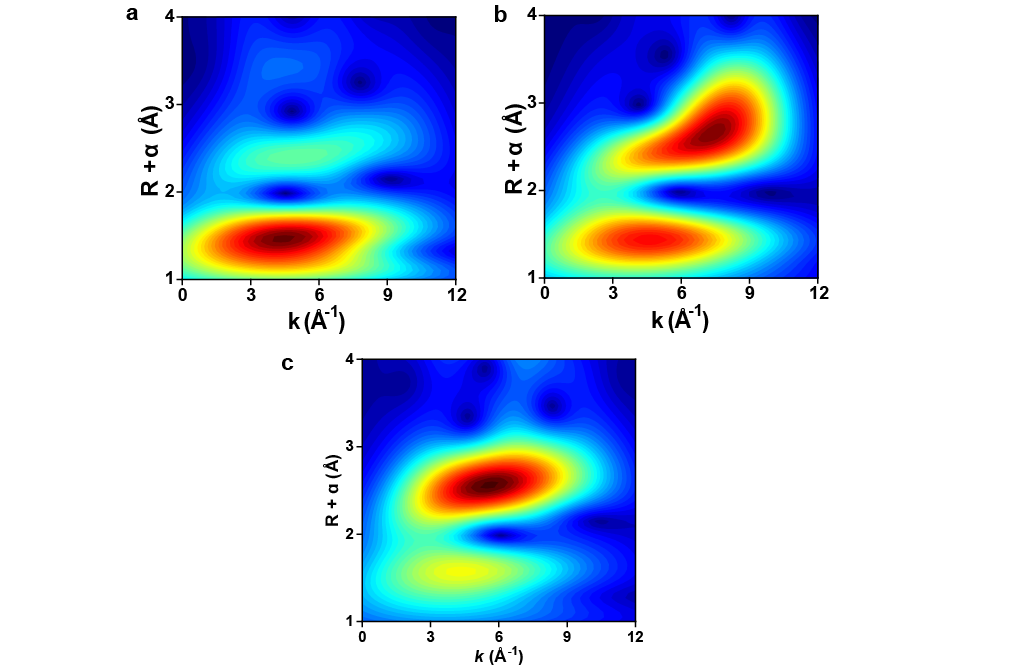


**Fig. S6** WT contour plots of Fe K-edge EXAFS spectra for a) FePc, b) Fe_2_O_3_, and c) Fe_3_O_4_.


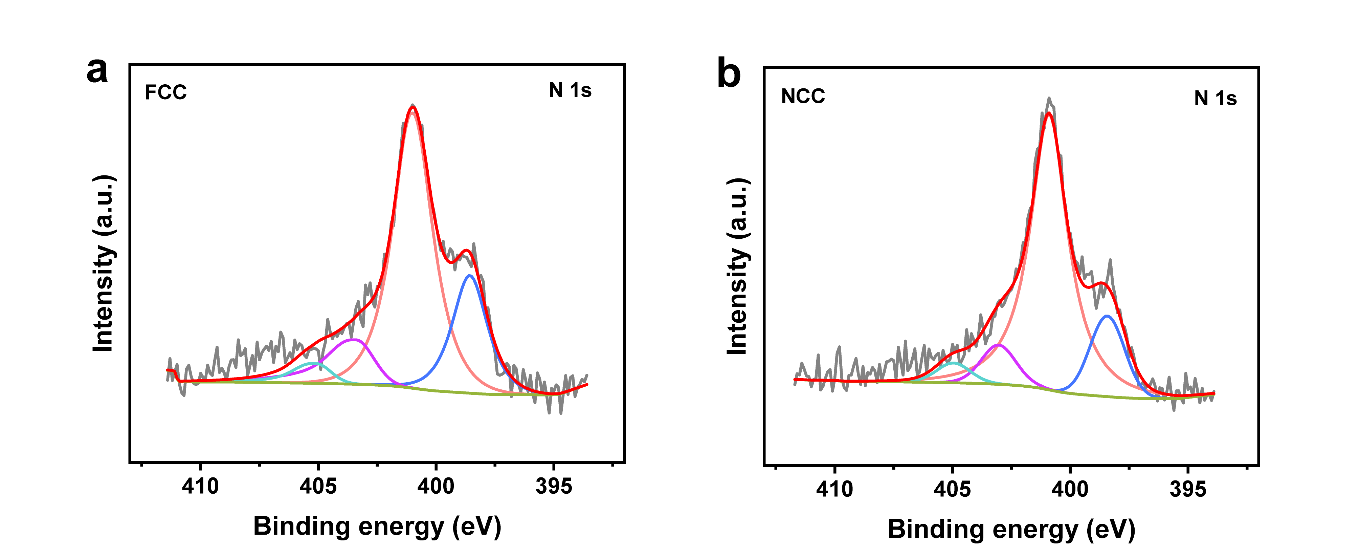


**Fig. S7** N 1s XPS spectra of a) FCC, and b) NCC.


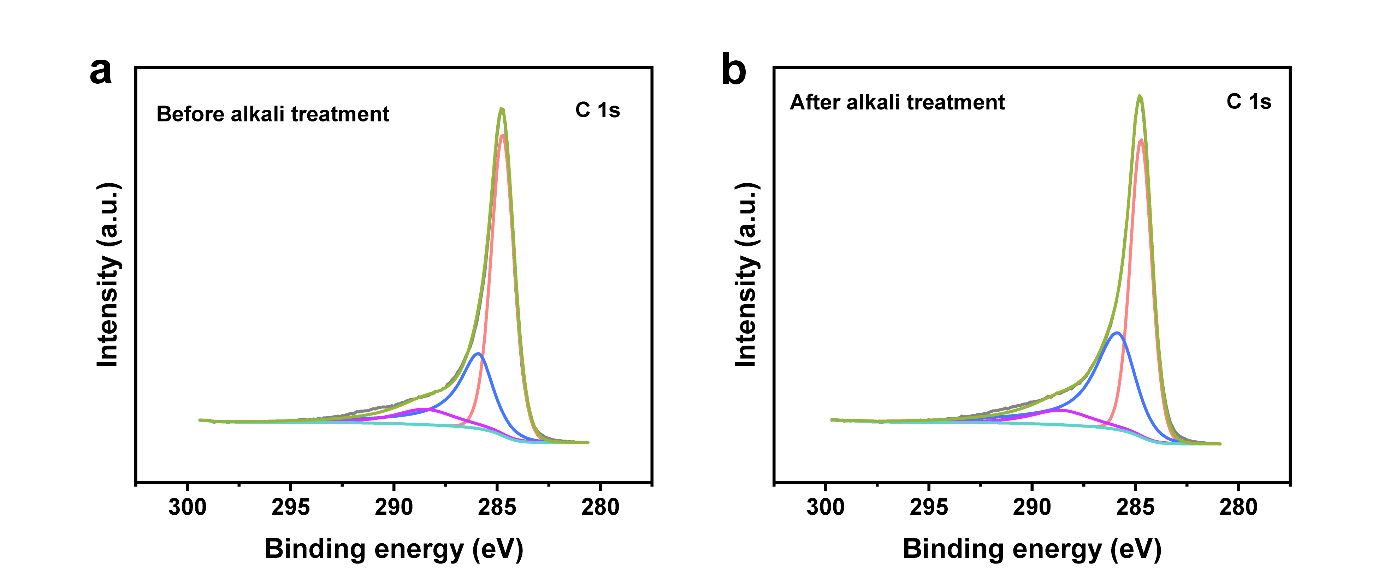


**Fig. S8** C 1s XPS spectra of FCC a) before and b) after alkali treatment.


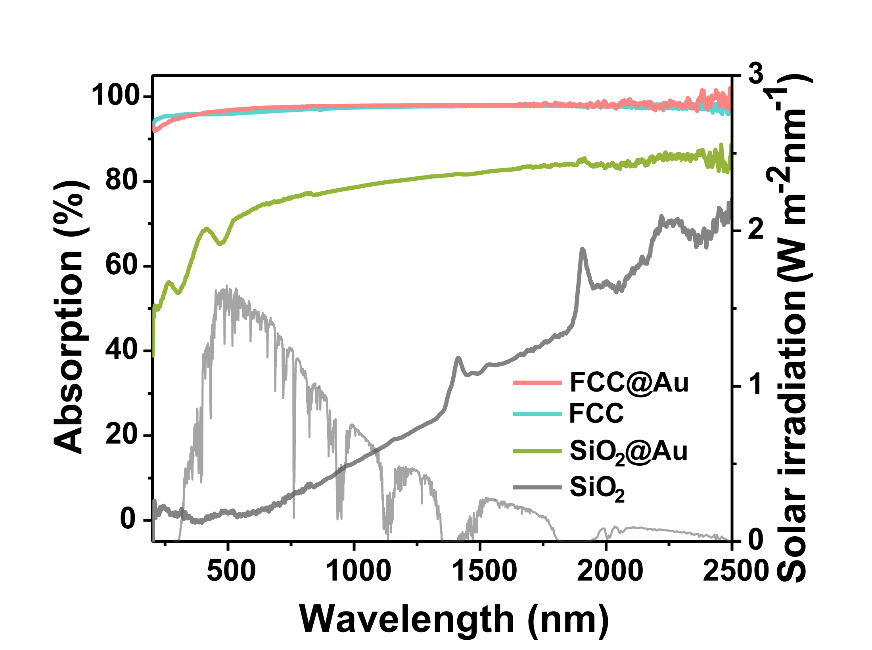


**Fig. S9** UV–vis–NIR absorption spectra of FCC, FCC@Au, SiO₂@Au, and SiO₂ powders, and standard AM1.5 solar spectrum.


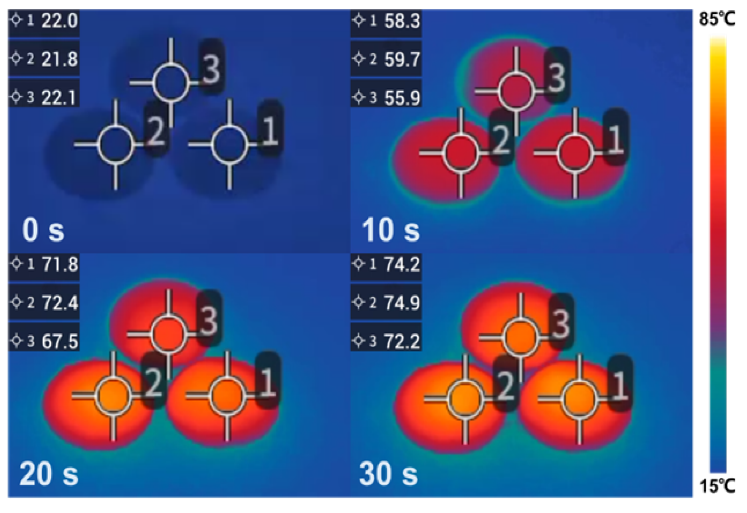


**Fig. S10** Infrared images of the sample powders when exposed to light in a dry state, in which FCC, FCC@Au and FCS are noted as 1, 2 and 3, respectively.


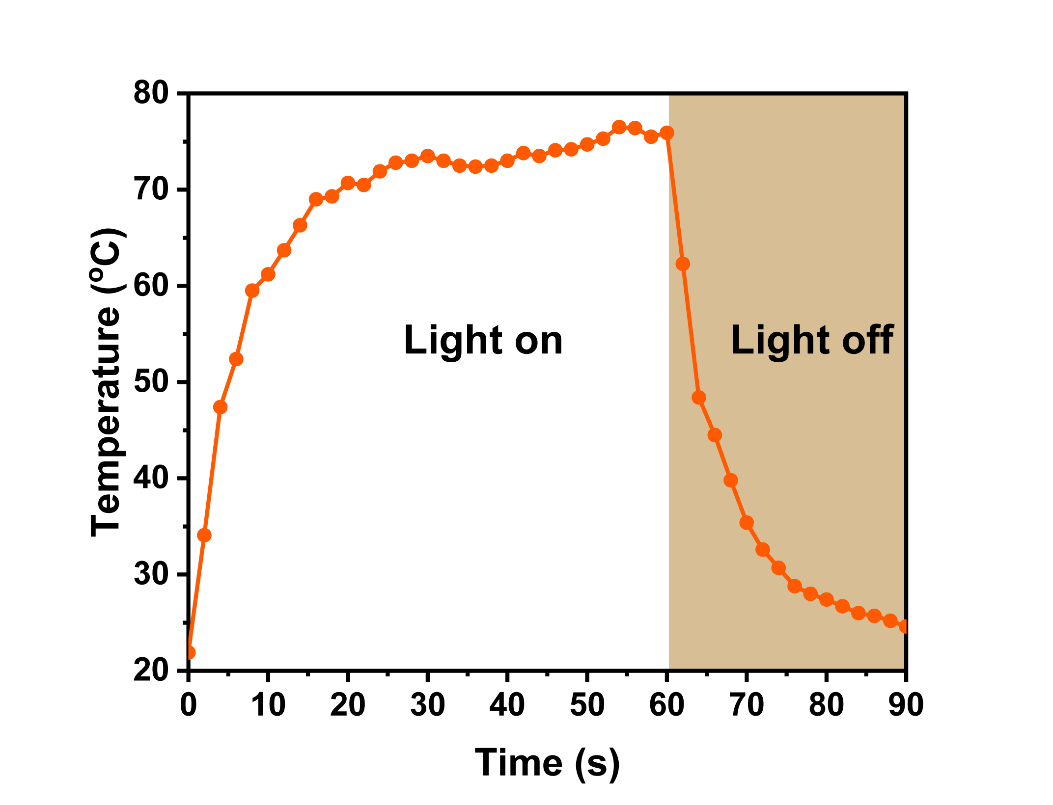


**Fig. S11** Temperature variation curve of dry FCC@Au powder when exposed to solar light.


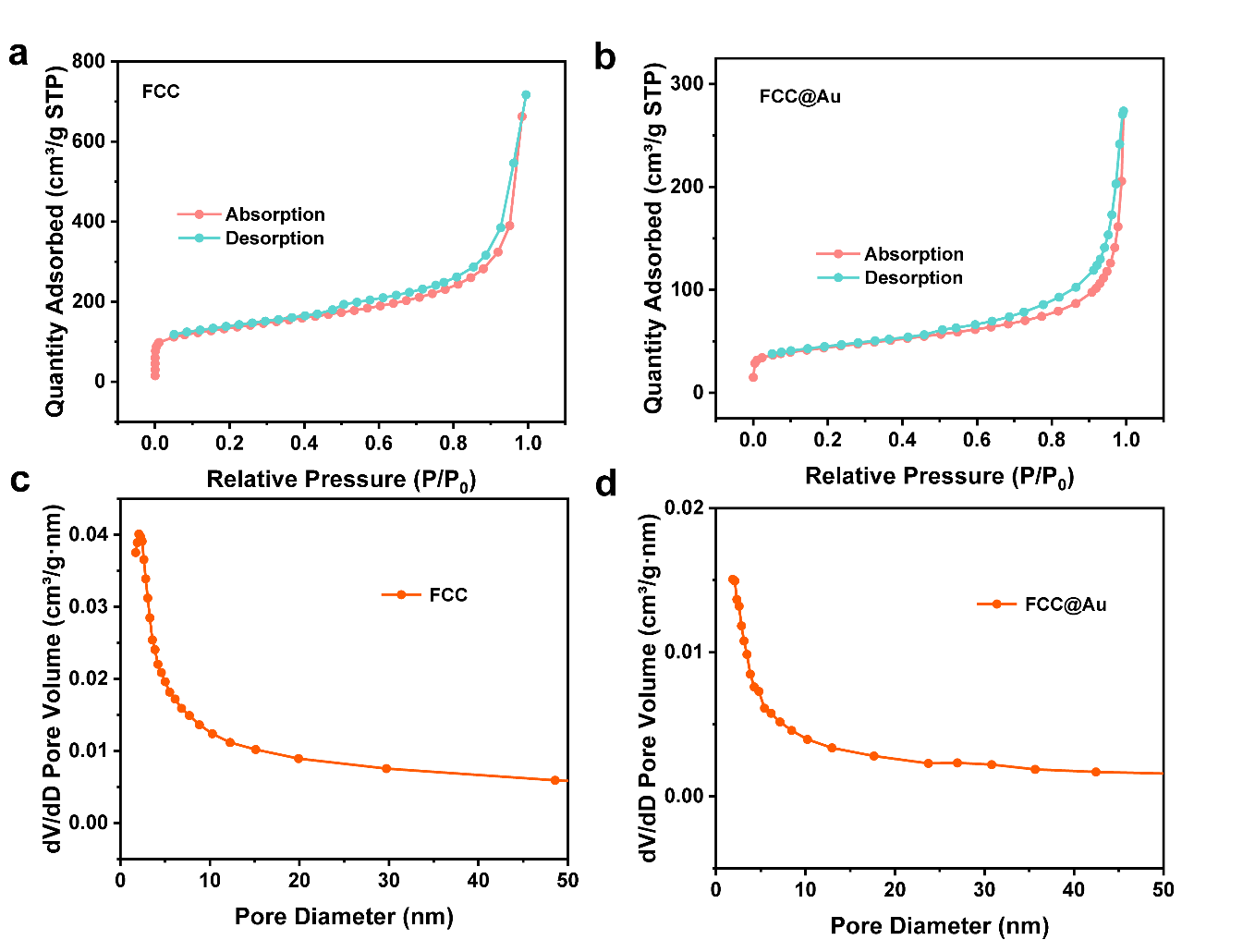


**Fig. S12** a, b) N_2_ adsorption-desorption curves, and c, d) pore size distribution curves of FCC, and FCC@Au.


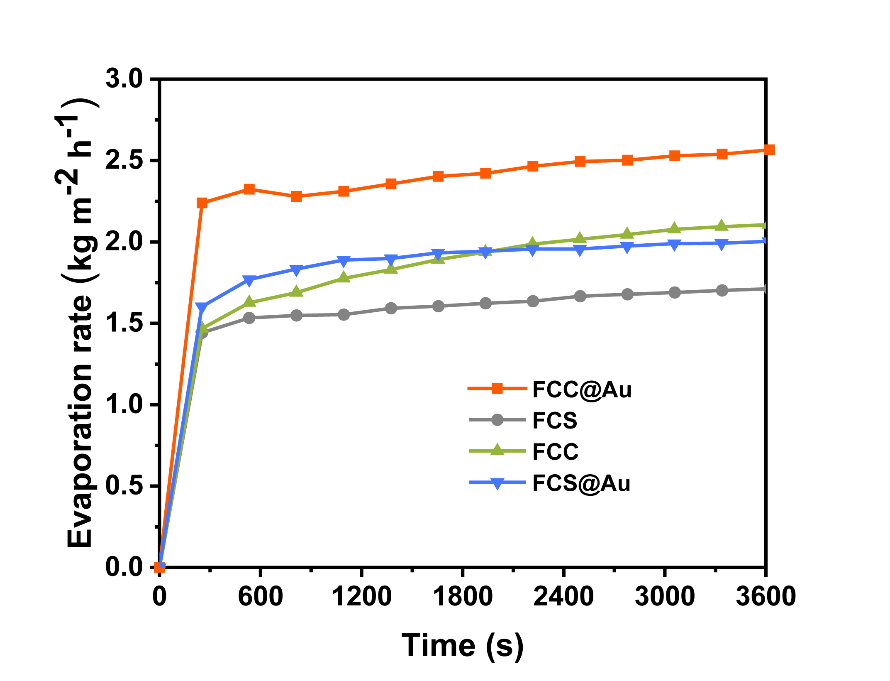


**Fig. S13** Plots of water evaporation rate of FCC@Au, FCC, FCS@Au, and FCS under 1-sun irradiation.


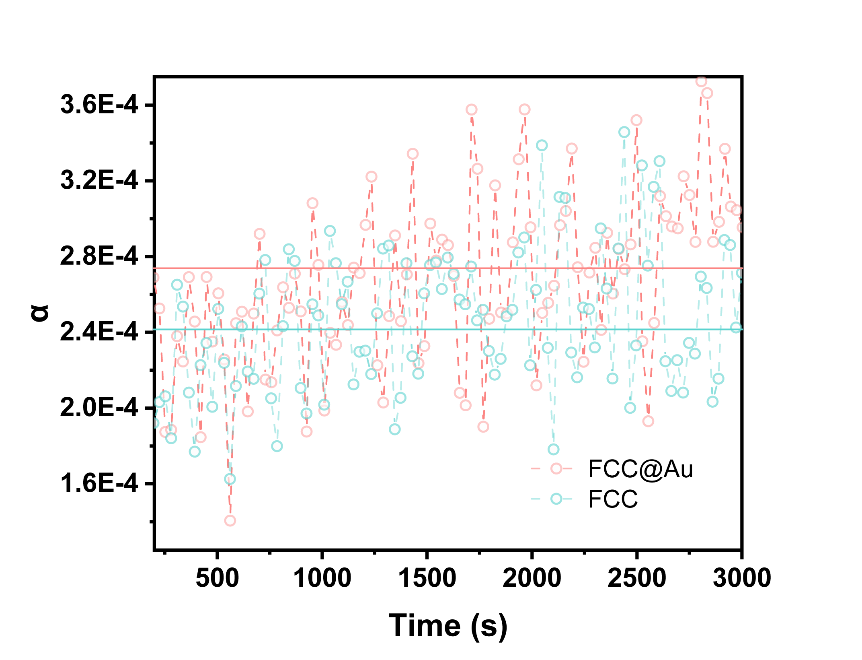


**Fig. S14** Calculated α values of FCC, and FCC@Au.


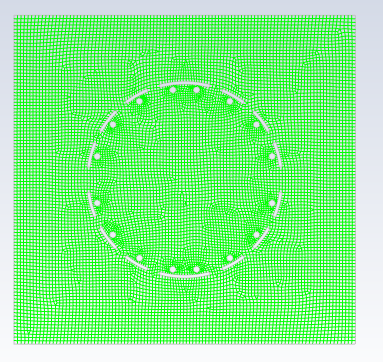


**Fig. S15** Schematic diagram of grid division for physical field simulation model.


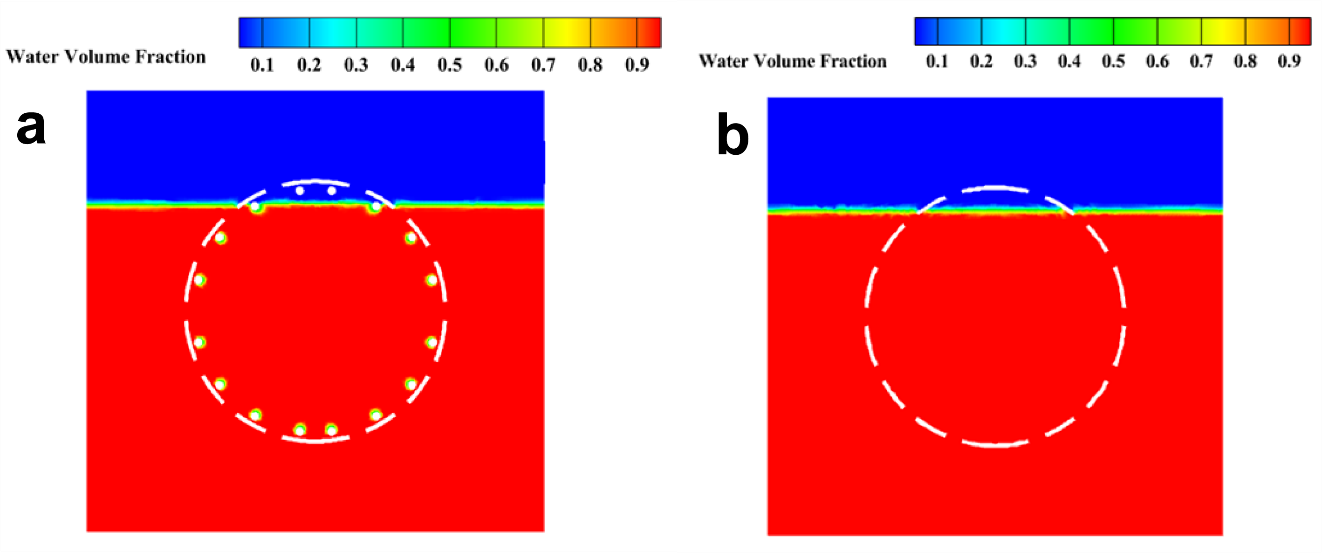


**Fig. S16** Phase diagrams of water and water vapor in a) FCC@Au and b) FCC.


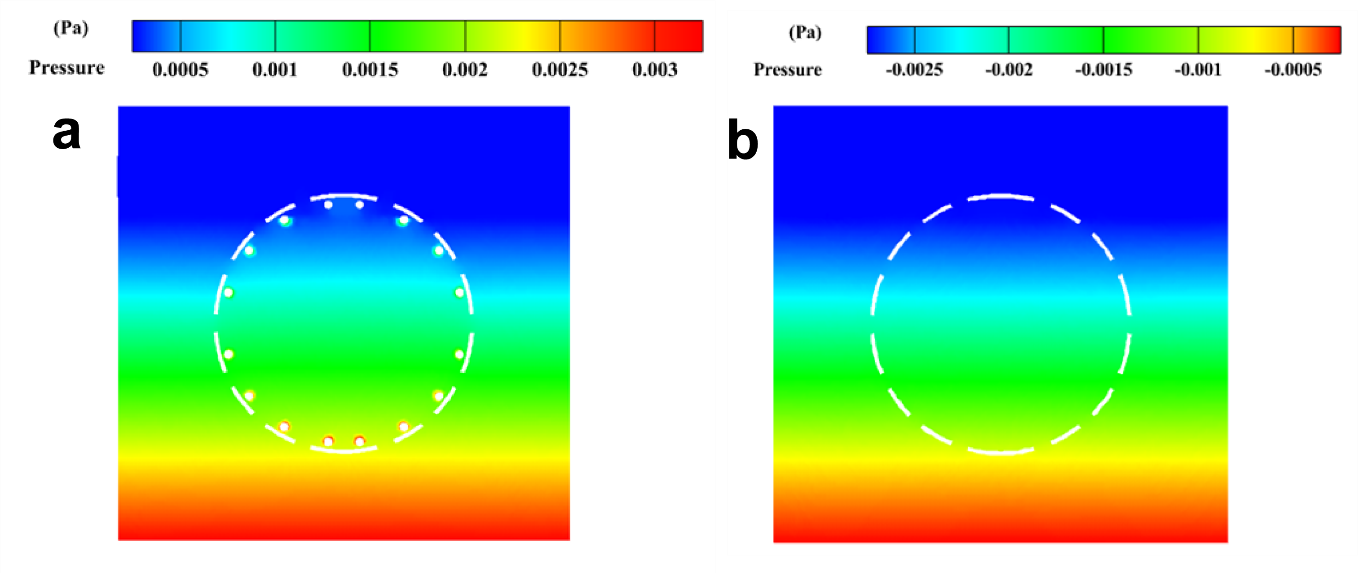


**Fig. S17** Pressure distribution maps of a) FCC@Au and b) FCC.


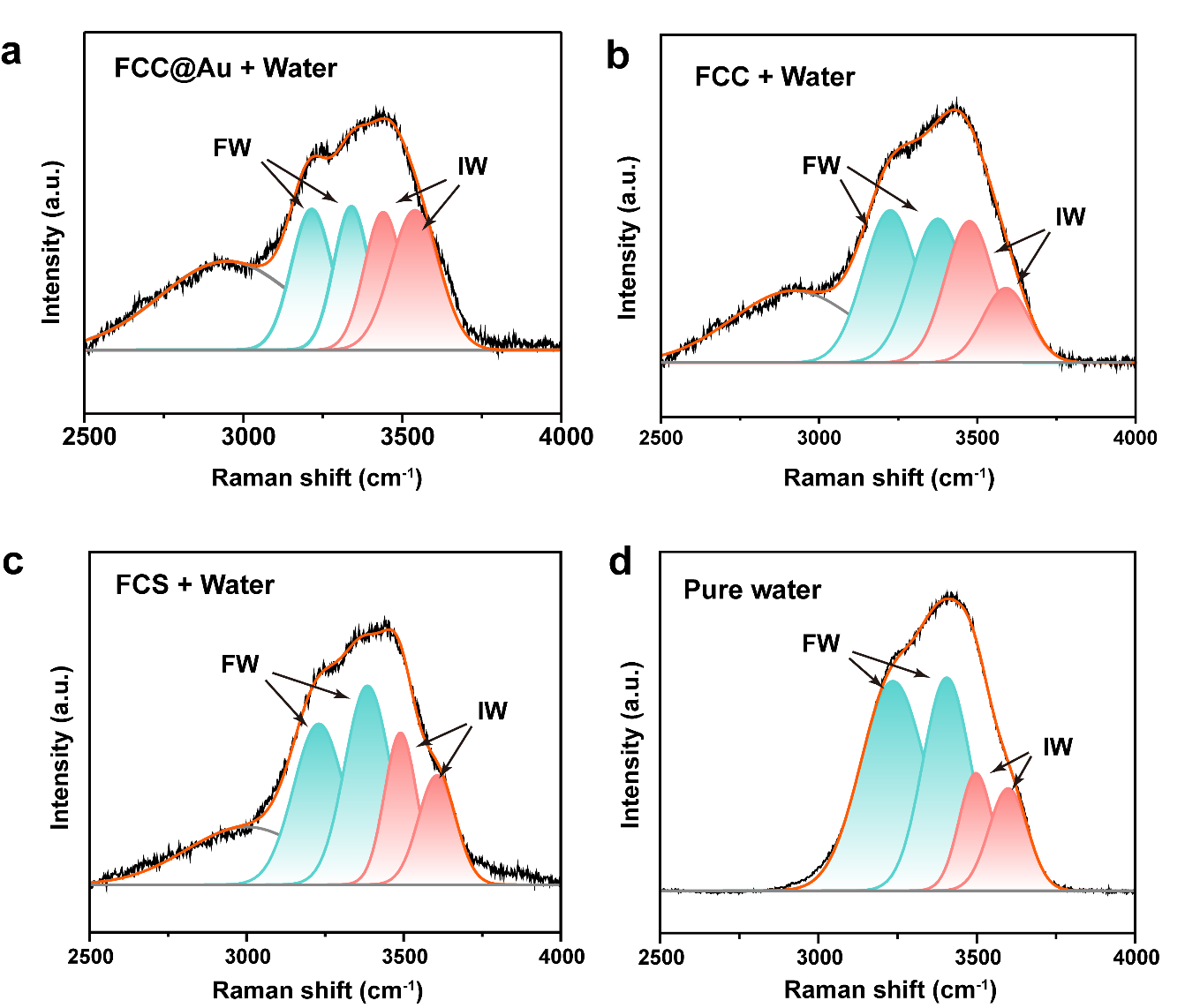


**Fig. S18** Raman spectra and deconvoluted O-H stretching peaks of water in a) FCC@Au, b) FCC, c) FCS, and d) pure water.


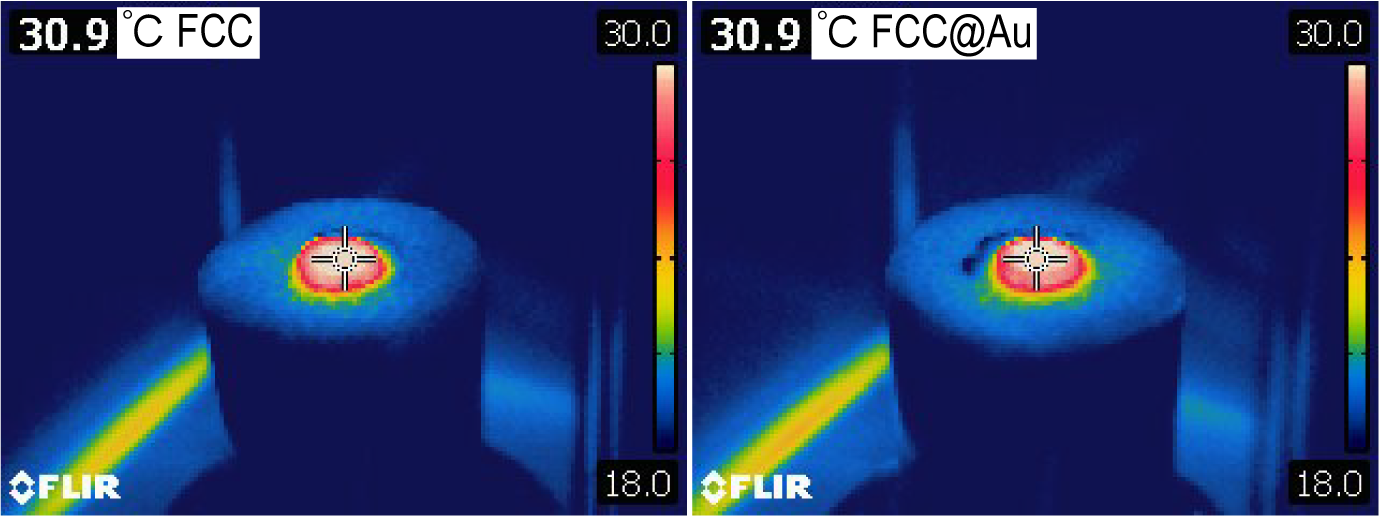


**Fig. S19** Stable infrared images of the planar evaporator made by FCC and FCC@Au under 1-sun irradiation.


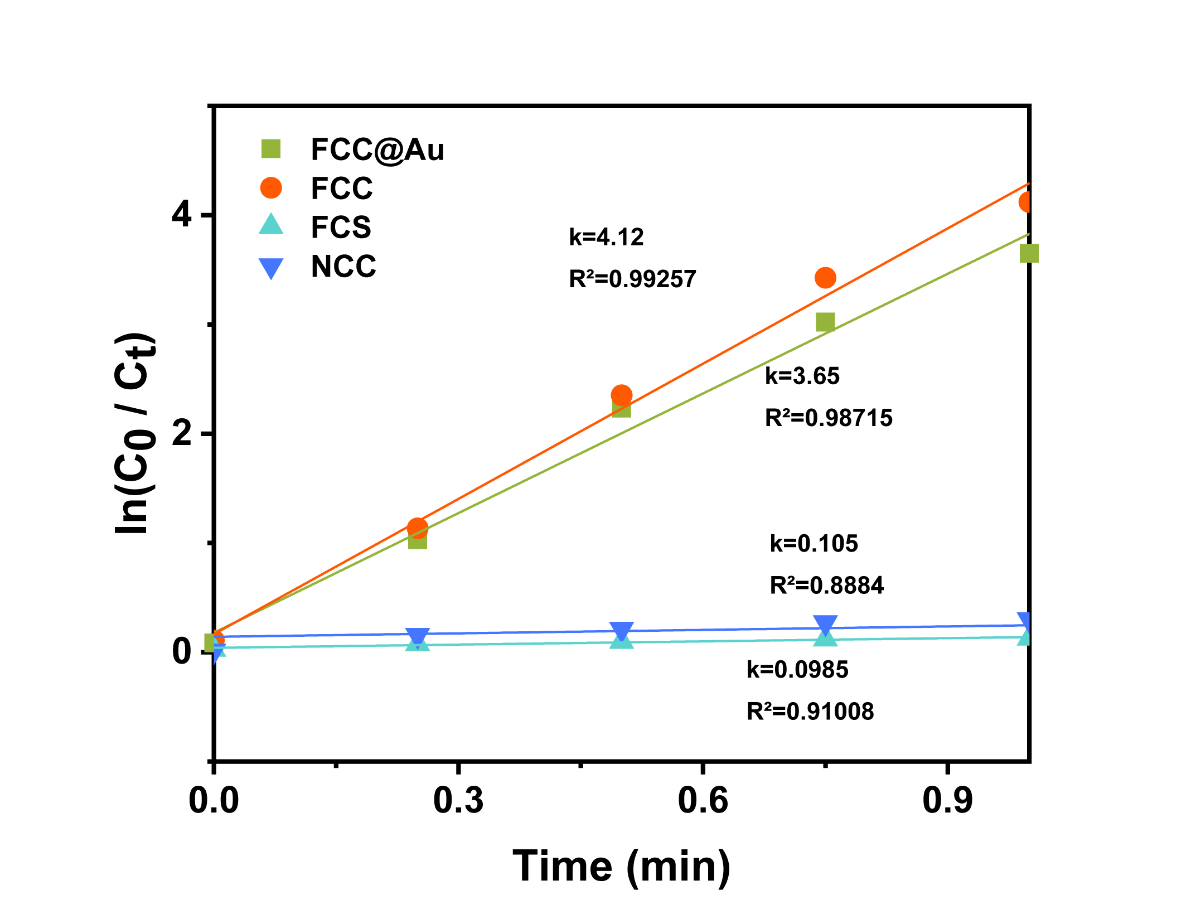


**Fig. S20** Pseudo-first-order kinetic fitting curves for the degradation of BPA over different samples.


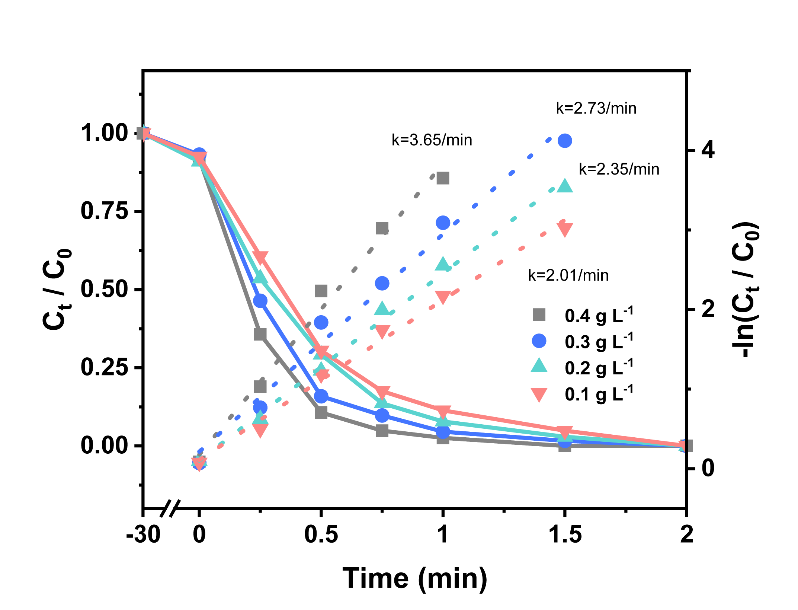


**Fig. S21** Degradation curves of BPA with different doses of PMS and corresponding apparent rate constants.


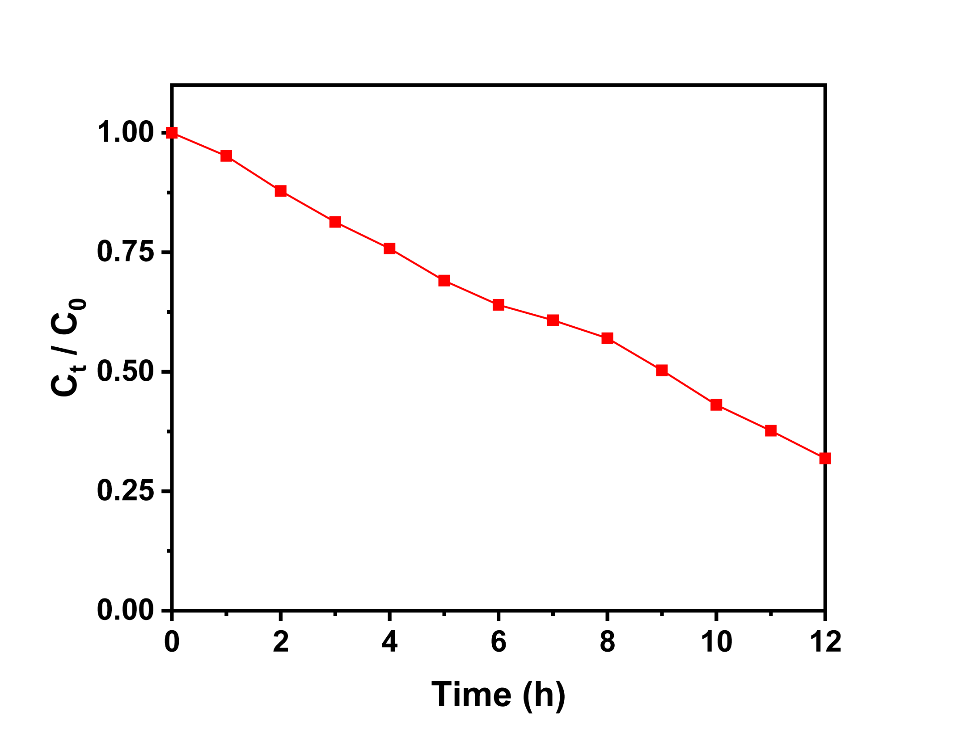


**Fig. S22** Degradation curve of BPA in the H-cell oxidation experiment.


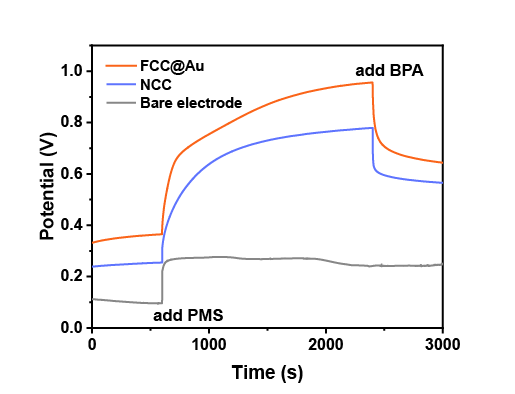


**Fig. S23** Open-circuit voltage curves of different samples.


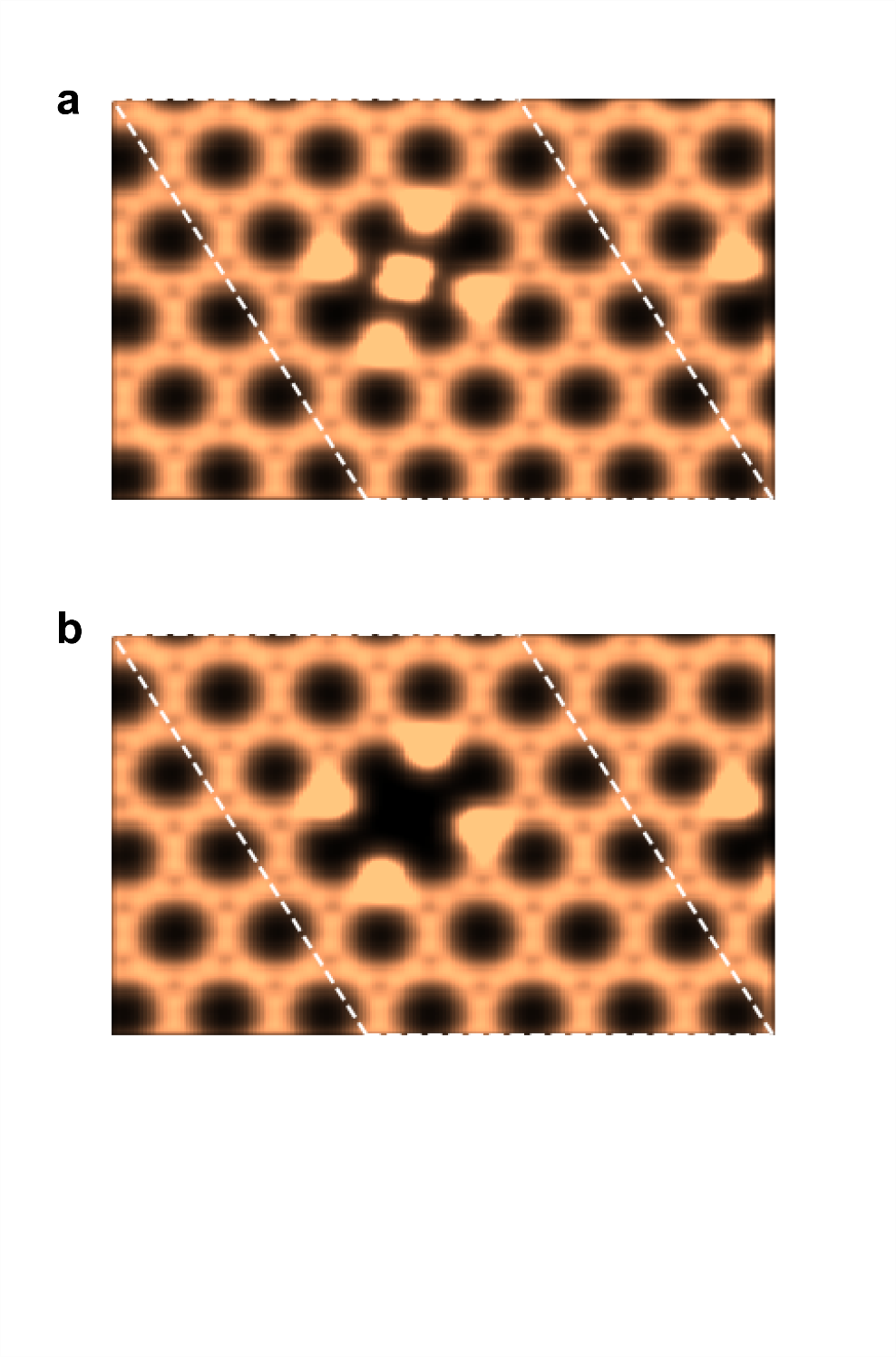


**Fig. S24** Calculated charge density maps highlighting the directional d–p electronic coupling in a) Fe–N₄ compared with the isotropic distribution in b) N₄–C.


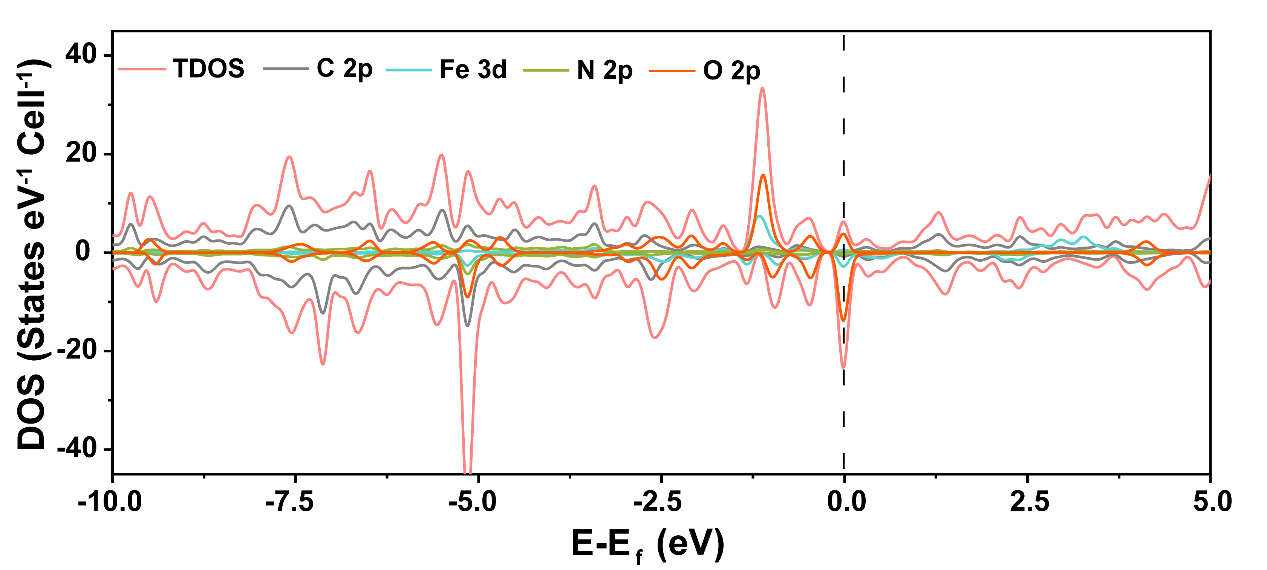


**Fig. S25** DOS of Fe-N_4_*PMS.


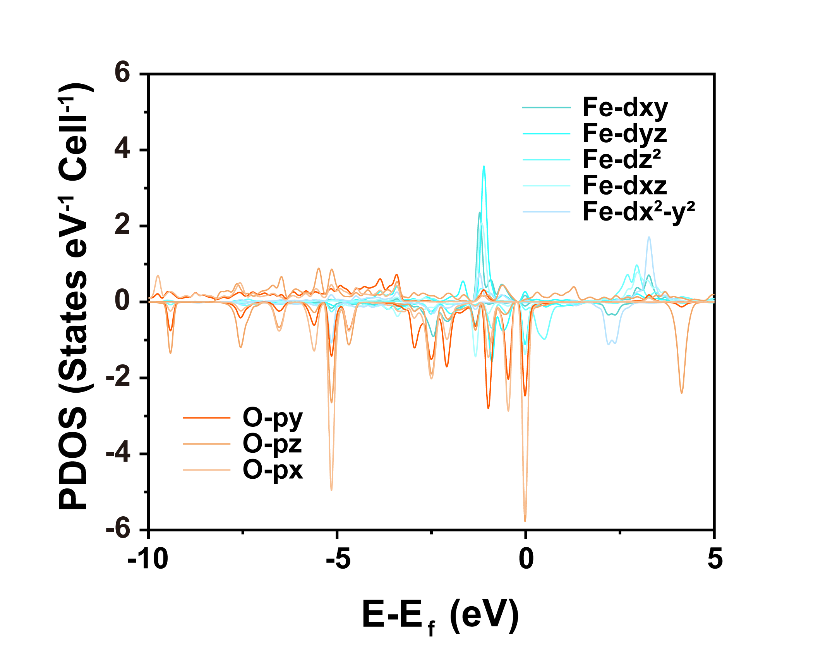


**Fig. S26** Orbital-resolved PDOS of Fe-N_4_*PMS.


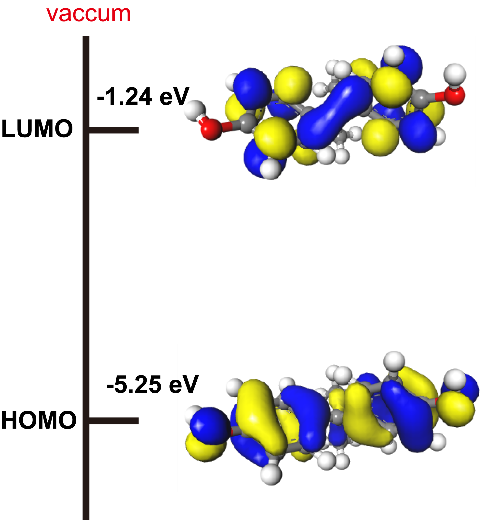


**Fig. S27** Frontier molecular orbitals diagram of BPA.


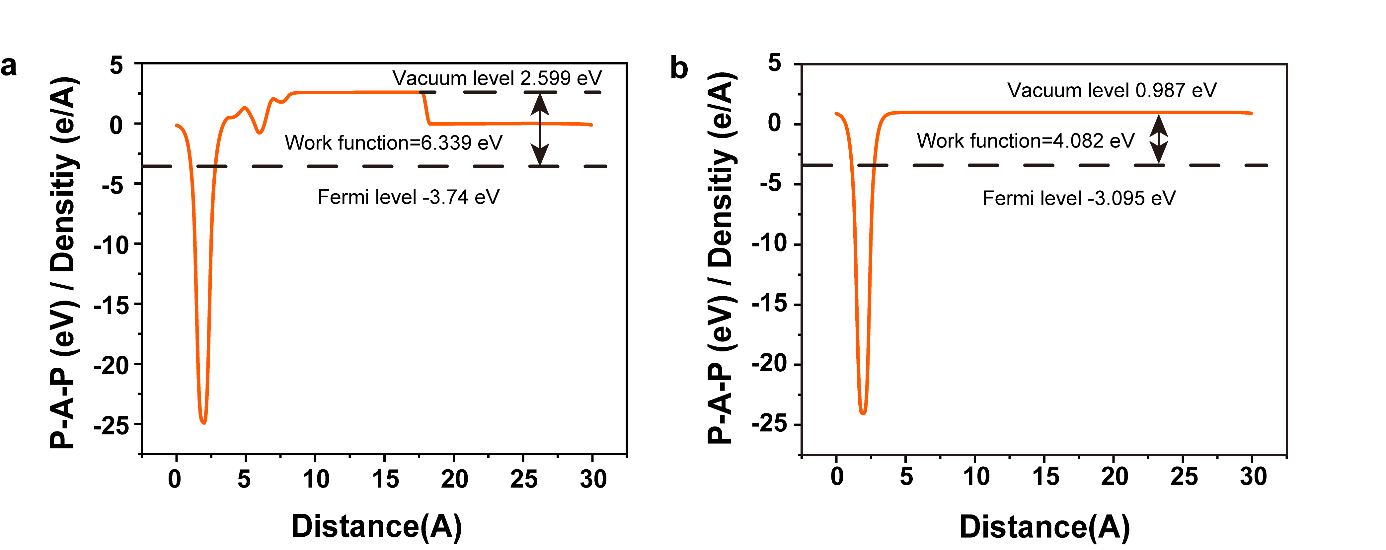


**Fig. S28** Planar-averaged electrostatic potential profiles of a) Fe-N_4_*PMS, and b) Fe-N_4_.


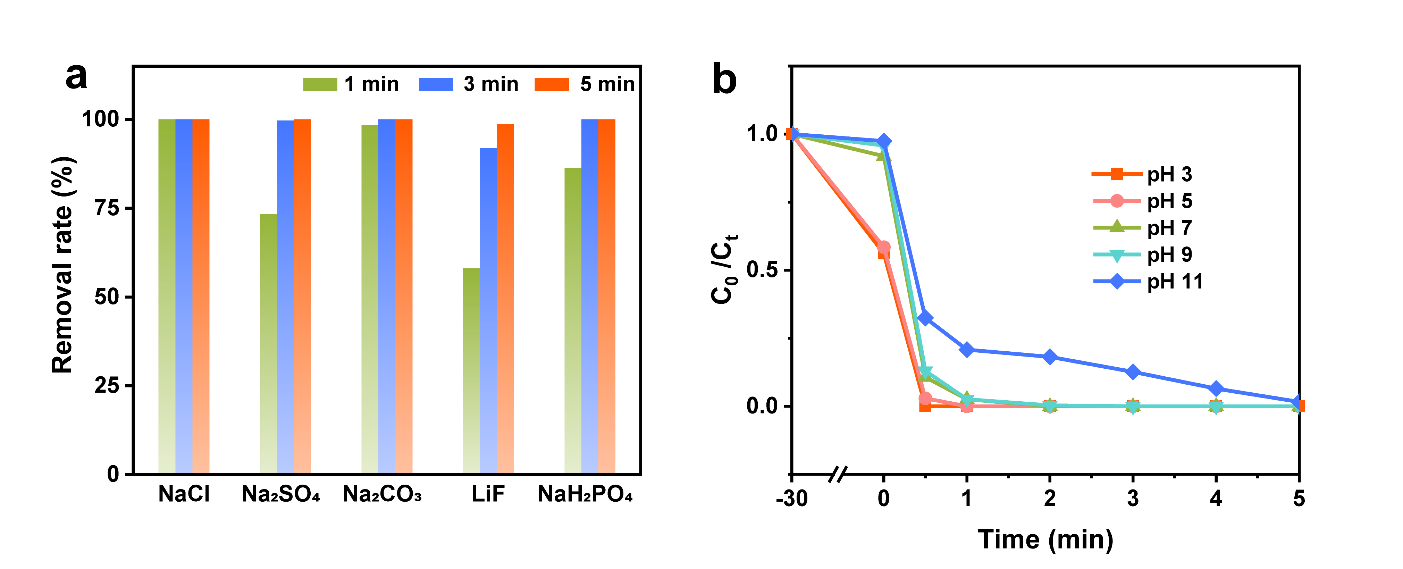


**Fig. S29** Degradation of BPA a) in different salt solutions (10 mM) and b) under different pH conditions.


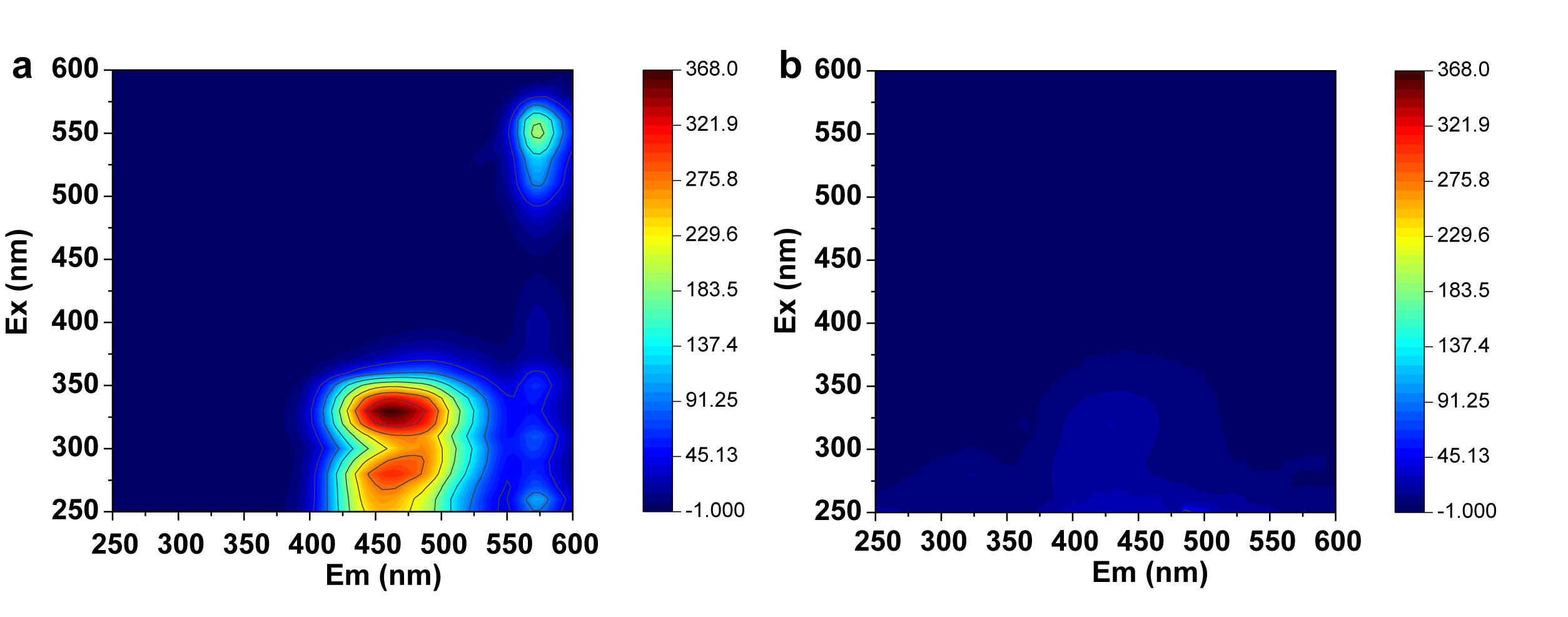


**Fig. S30** 3D fluorescence spectra of a) mixed pollutants and b) after degradation.


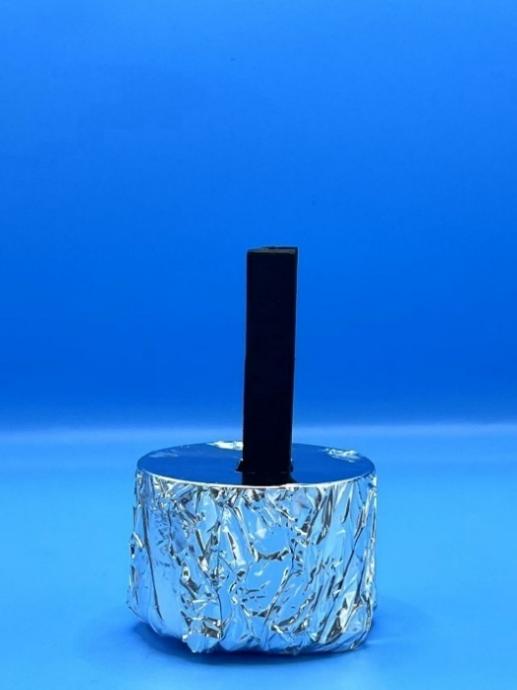


**Fig. S31** Photograph of the laboratory-based solar-thermal water evaporator.


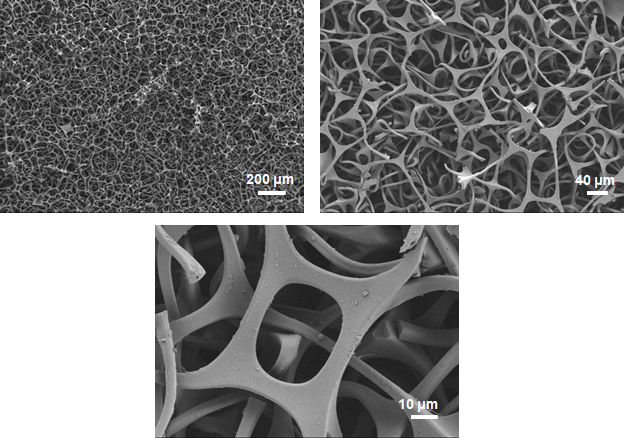


**Fig. S32** SEM images of polyurethane foam.


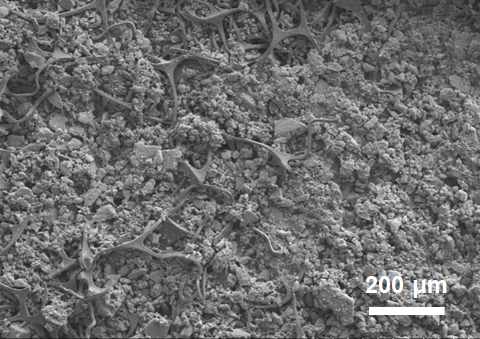


**Fig. S33** SEM image of the polyurethane foam loaded with FCC@Au.


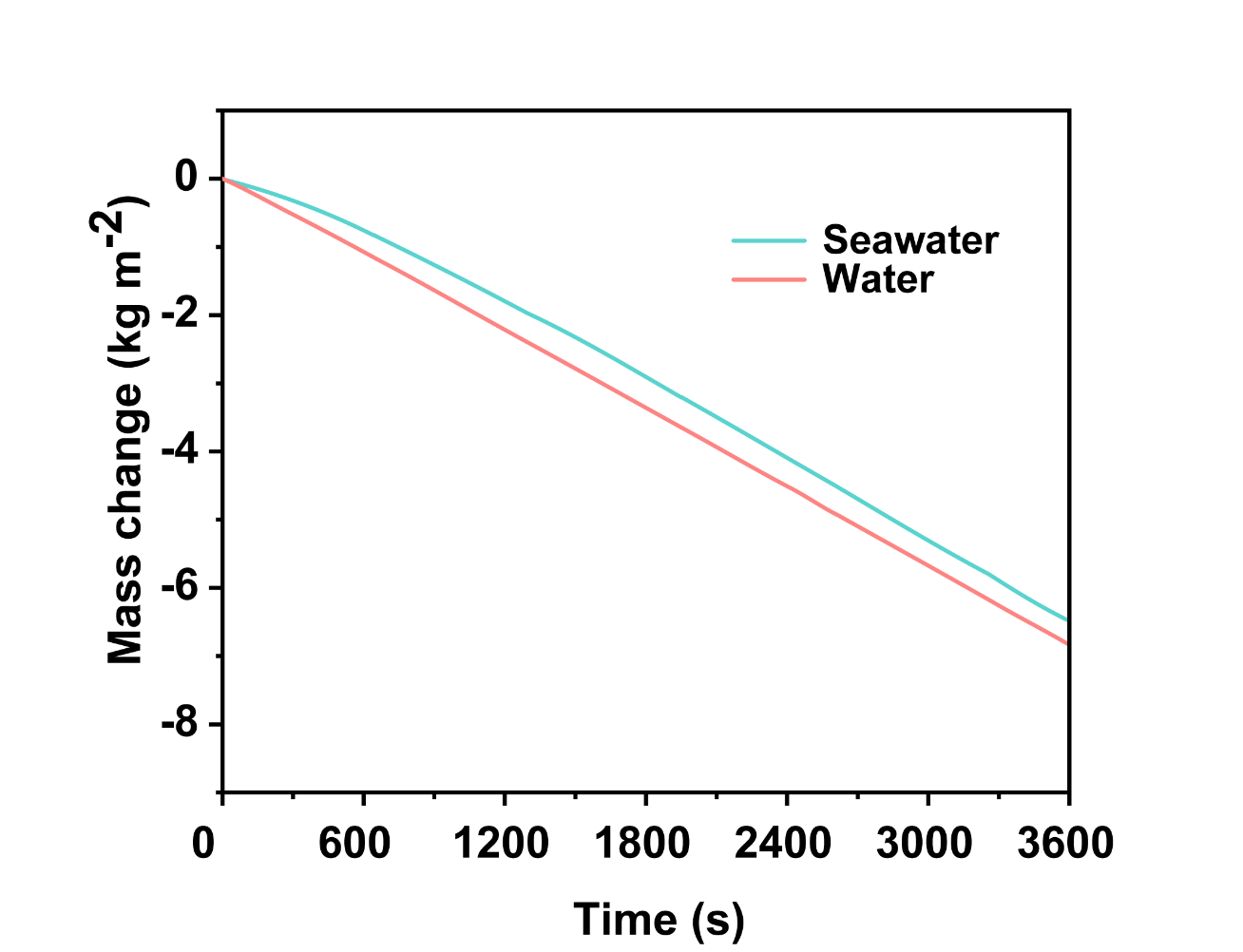


**Fig. S34** Evaporation mass change curves of the FCC@Au/PU evaporator in pure water and seawater.


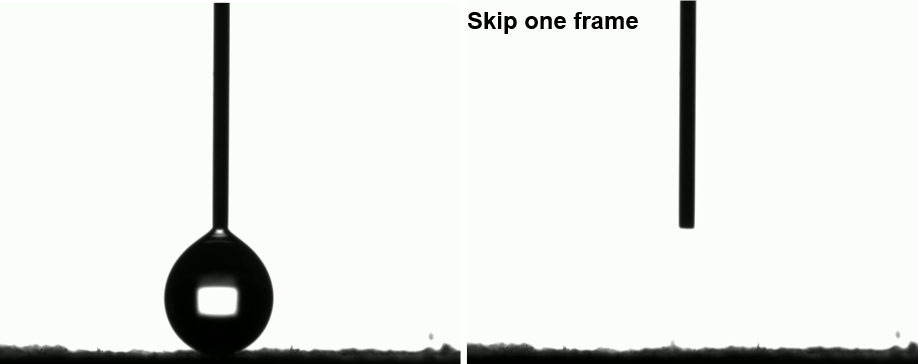


**Fig. S35** Contact angle test screenshot of the polyurethane foam loaded with FCC@Au.


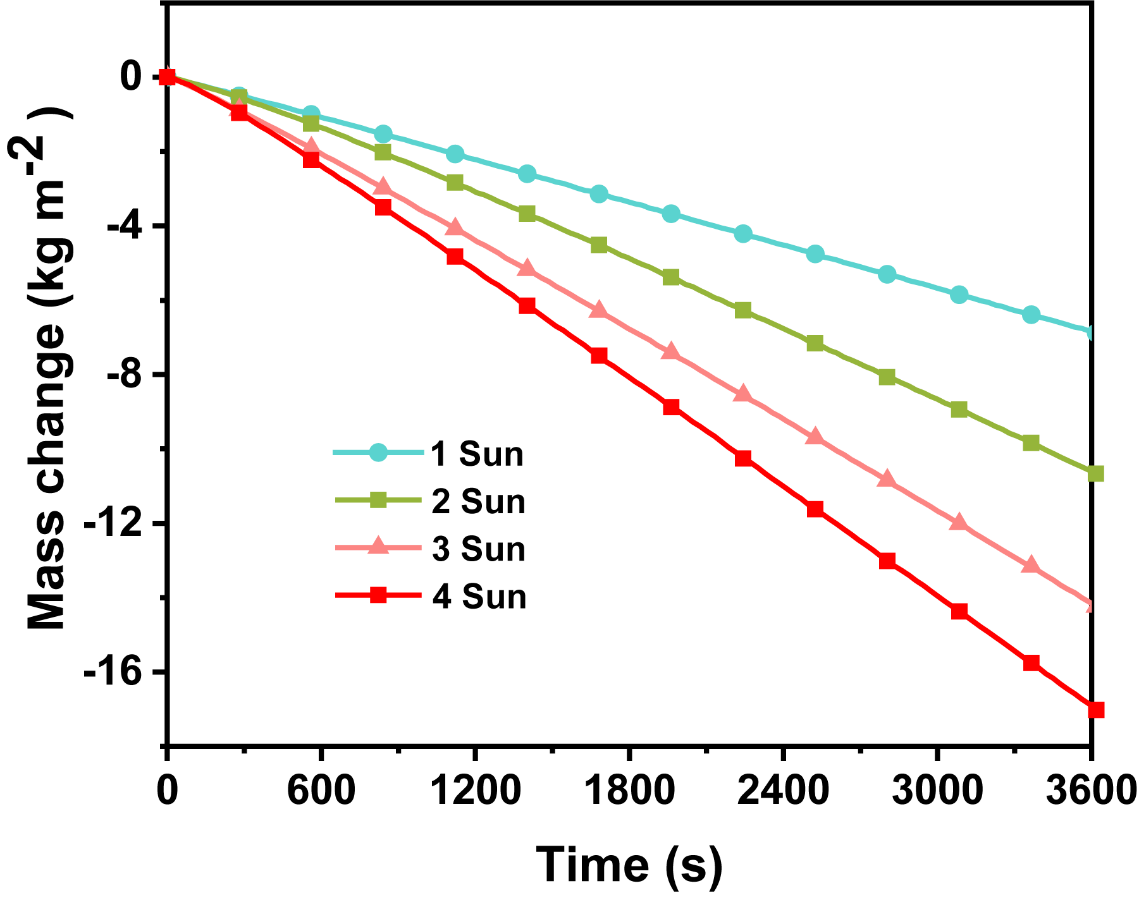


**Fig. S36** Mass change curves of the PU foam loaded with FCC@Au under different solar intensities.


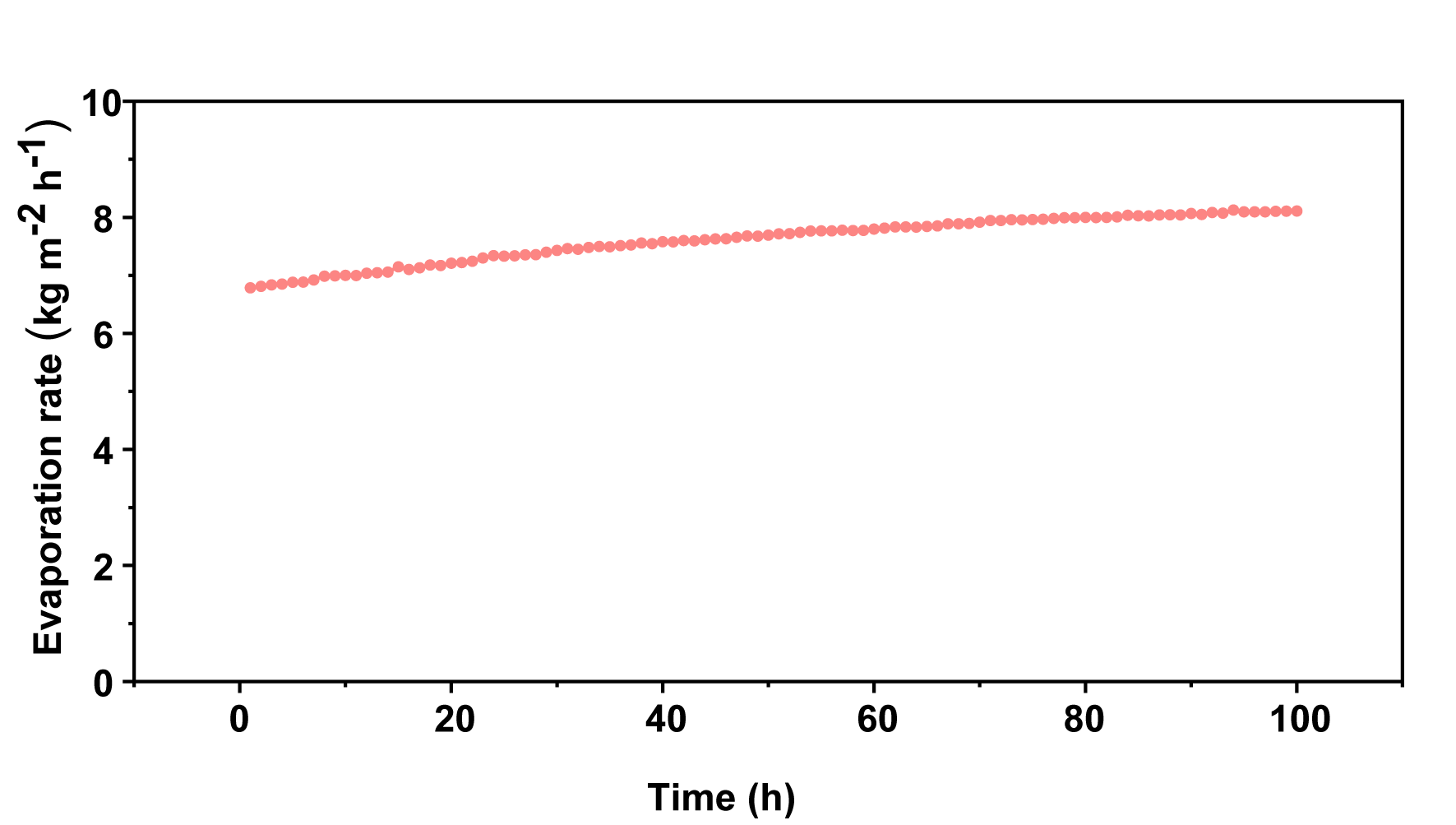


**Fig. S37** 100-hour long-cycle test of the FCC@Au/PU evaporator.


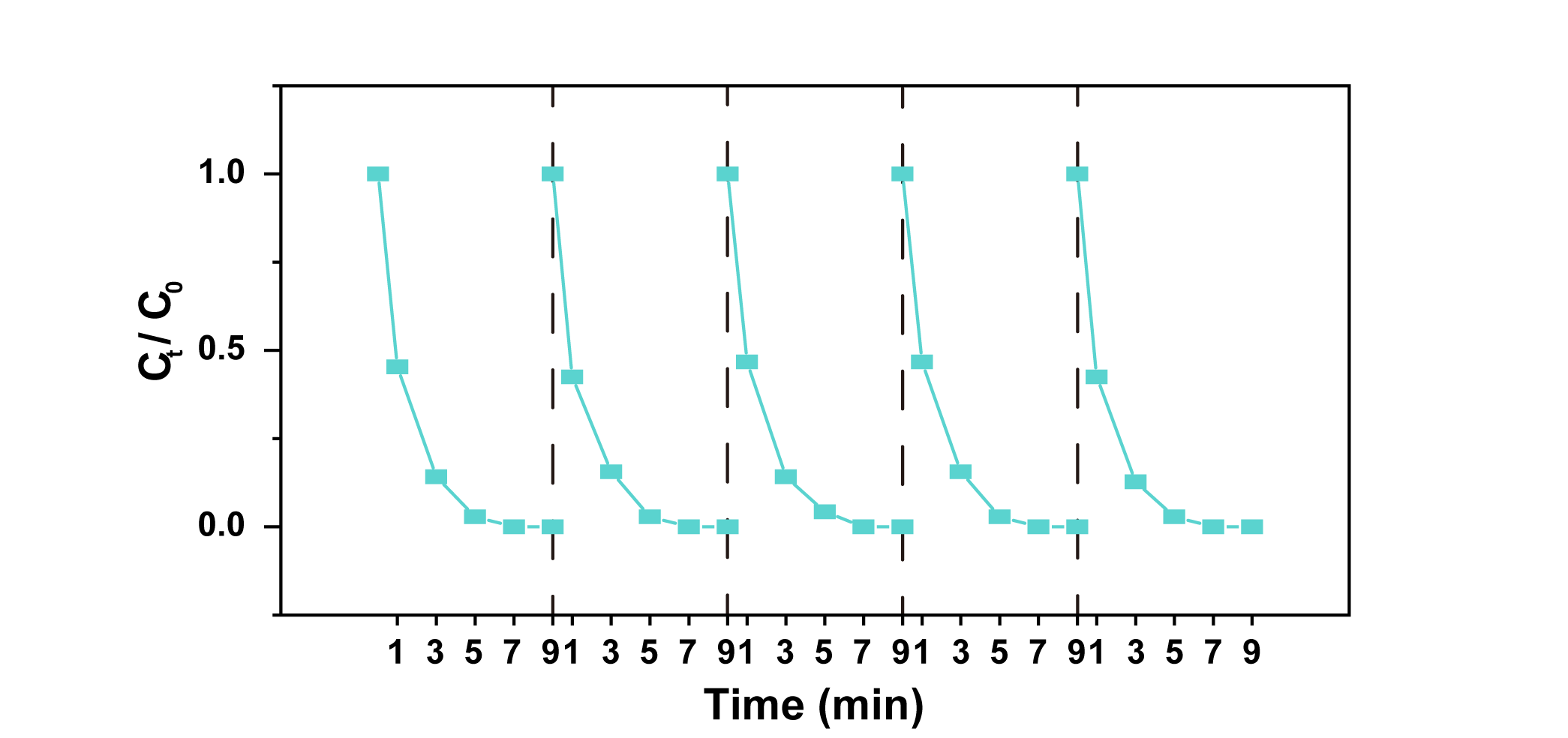


**Fig. S38** Catalytic capacity cycling test of FCC@Au/PU evaporator.


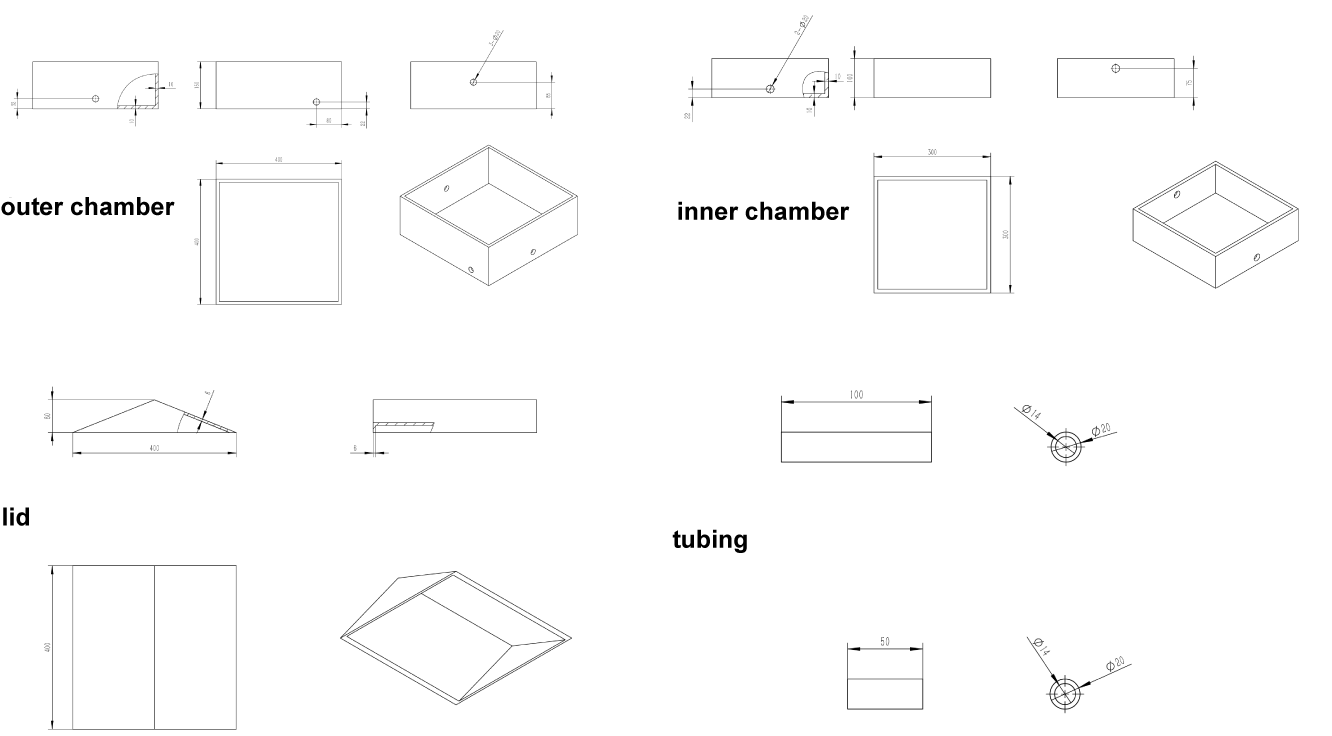


**Fig. S39** Schematic illustration of the simplified outdoor experimental setup designed to evaluate the practical application potential of the evaporator. The system consists of a sealed container, inlet, and 2 different types outlet pipes for water supply and collection, and a solar-absorbing evaporator mounted on a floating support.


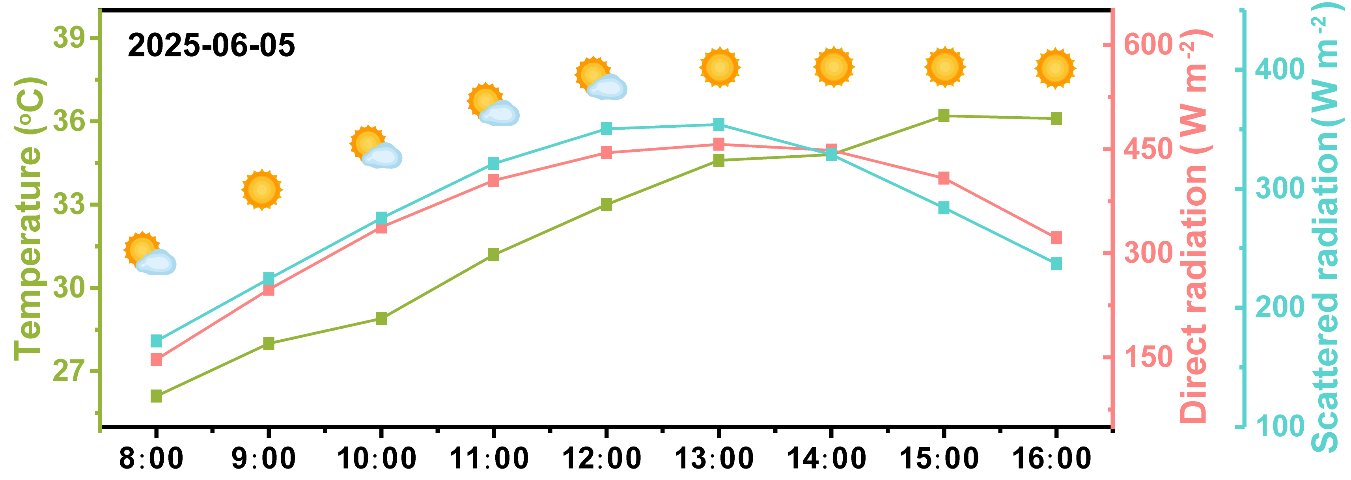


**Fig. S40** Real-time temperature and solar irradiation profiles recorded during the continuous operation.


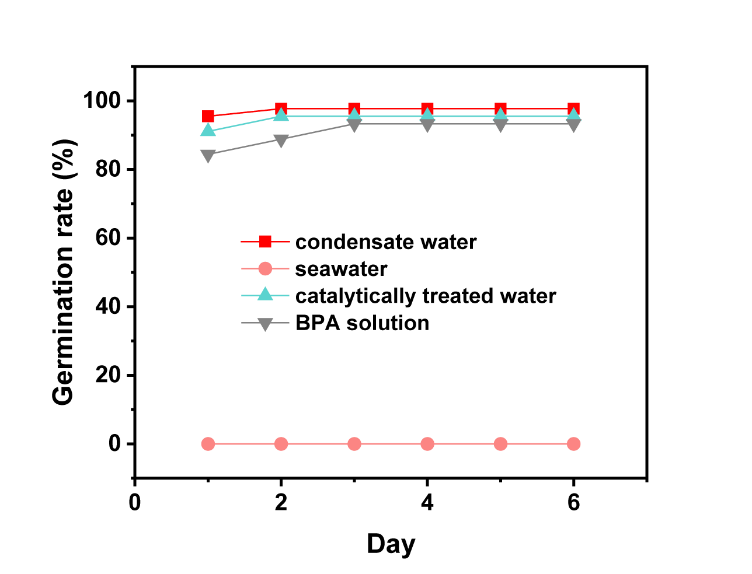


**Fig. S41** Germination rates using different water sources.


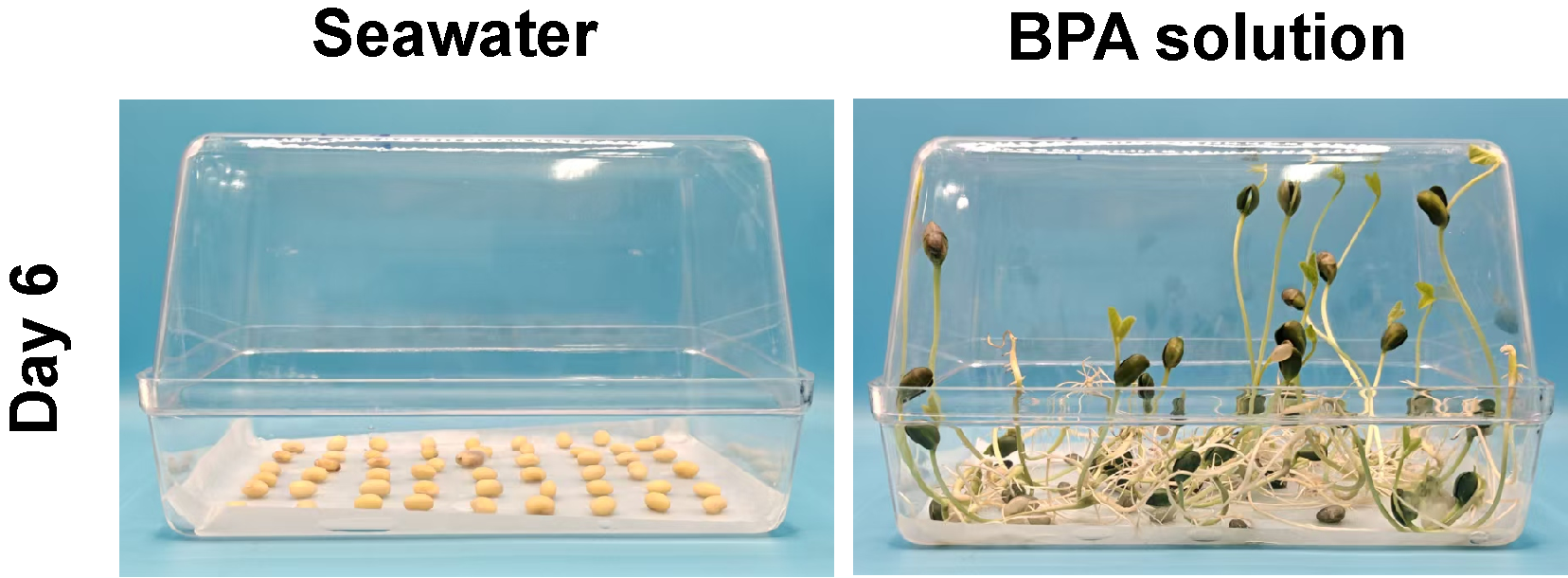


**Fig. S42** Images of seed germination tests using seawater and BPA solution over 6 days.

**Supplementary Tables**

**Table S1** Water evaporation and pollutant degradation abilities of different materials

| NO. | Materials | Evaporation capacity  (kg m^−2^ h^−1^) | m(PMS)/m(BPA) | K_obs_/m(catalyst)  (L g^-1^ min^-1^) | Ref. |
| --- | --- | --- | --- | --- | --- |
| 1 | MXene/Au@Cu_2−x_S | 2.023 |  |  | [S14] |
| 2 | PDA/GF/PVA/PVP | 1.60 |  |  | [S15] |
| 3 | Hanging fabric | 2.6 |  |  | [S16] |
| 4 | PPy nanosheets | 1.38 |  |  | [S17] |
| 5 | Bridge arch resin | 1.64 |  |  | [S18] |
| 6 | PPy membranes | 2.03 |  |  | [S19] |
| 7 | Ag@MXene/PAN | 2.08 |  |  | [S20] |
| 8 | GO/CNT@PIL | 1.87 |  |  | [S21] |
| 9 | CNT@AAO | 1.47 |  |  | [S22] |
| 10 | Ba_x_Sr_1-x_Co_y_Fe_1-y_O_3-δ_ |  | 12.5 | 3.08 | [S23] |
|  |  |  | 25 | 2.41 |  |
|  |  |  | 37.5 | 3.21 |  |
|  |  |  | 50 | 2.4 |  |
| 11 | MXene-MOFs |  | 5 | 9.44 | [S24] |
| 12 | Co-NC-PS |  | 5 | 0.32 | [S25] |
|  |  |  | 10 | 3.79 |  |
|  |  |  | 15 | 12.9 |  |
|  |  |  | 25 | 13.49 |  |
| 13 | N and O co-doped porous carbon |  | 1.5 | 0.19 | [S26] |
|  |  |  | 3 | 1.12 |  |
|  |  |  | 4.5 | 1.5 |  |
|  |  |  | 9 | 5.57 |  |
| 14 | Fe-N_2_B_4_ |  | 7.5 | 0.39 | [S27] |
|  |  |  | 15 | 0.55 |  |
|  |  |  | 30 | 1.24 |  |
|  |  |  | 60 | 1.81 |  |
| 15 | FCC@Au |  | 10 | 100.5 | This work |
|  |  |  | 20 | 117.5 |  |
|  |  |  | 30 | 136.5 |  |
|  |  |  | 40 | 182.5 |  |

**Table S2** Structural parameters obtained from Fe K-edge EXAFS fitting for Fe foil and FCC.

|  | S_0_^2^ | | shell | CN^a^ | R(Å)^b^ | σ^2^ (10^-3^ Å^2^)^c^ | Δ*E*_0_^d^ | R factor^e^ |
| --- | --- | --- | --- | --- | --- | --- | --- | --- |
| Fe foil | | 0.98 | Fe-Fe | 8 | 2.47±0.01 | 7.5±0.7 | 5.40±2.48 | 0.0094 |
|  | |  | Fe-Fe | 6 | 2.84±0.01 | 6.9±1.4 |  |  |
| FCC | | 0.98 | Fe-N | 4.1 | 2.09±0.01 | 1.4±0.1 | 8.50±0.01 | 0.0177 |

^a^ Coordination numbers; ^b^ bond distance; ^c^ Debye-Waller factors; ^d^ the inner potential correction. ^e^ goodness of fit.

**Table S3** Water evaporation and pollutant degradation abilities of different materials

| NO. | Materials | Evaporation capacity | | Pollutants for degradation | Degradation ability  Powder Loaded | | Ref. |
| --- | --- | --- | --- | --- | --- | --- | --- |
| 1 | PEG foam | | 4.32 kg m^–2^ h^–1^ |  |  |  | [S28] |
| 2 | PNDA-PVA | | 8.7 kg m^–2^ h^–1^ |  |  |  | [S29] |
| 3 | Metal–phenolic | | 3.3 kg m^–2^ h^–1^ |  |  |  | [S30] |
| 4 | Plasmonic sponge | | 2.02 kg m^–2^ h^–1^ |  |  |  | [S31] |
| 5 | Hanging fabric | | 2.6 kg m^–2^ h^–1^ |  |  |  | [S16] |
| 6 | FeNC-800 | |  | 20 ppm BPA | K_obs_=22.74 min^-1^ |  | [S32] |
| 7 | MCoO@Co-N-C | |  | 20 ppm BPA | K_obs_=0.472 min^-1^ |  | [S24] |
| 8 | Fe-N_2_B_4_ | |  | 20 ppm BPA | K_obs_=0.25 min^-1^ |  | [S27] |
| 9 | PPy/RGO/Co_3_(PO_4_)_2_ | | 2.08 kg m^–2^ h^–1^ | 50 ppm RhB |  | Complete degradation within 6 mins | [S33] |
| 10 | Co-NCNT/CF | | 3.85 kg m^–2^ h^–1^ | 50 ppm PE |  | 85% degradation within 1 h | [S24] |
| 11 | MnO_2_/CNT/wood | | 2.74 kg m^–2^ h^–1^ | 20 ppm MO |  | K_obs_=0.201 min^-1^ | [S35] |
| 12 | CoFe_2_O_4_/CNT/ bamboo fabrics | | 2.72 kg m^–2^ h^–1^ | 10 ppm BPA |  | K_obs_=0.149 min^-1^ | [S36] |

**Supplementary References**

1. R. Marek, J. Straub. Analysis of the evaporation coefficient and the condensation coefficient of water. Int. J. Heat Mass Transfer. **44**(1), 39-53 (2001). <https://doi.org/https://doi.org/10.1016/S0017-9310(00)00086-7>
2. C. W. Hirt, B. D. Nichols. Volume of fluid (vof) method for the dynamics of free boundaries. J. Comput. Phys. **39**(1), 201-225 (1981). <https://doi.org/https://doi.org/10.1016/0021-9991(81)90145-5>
3. Z. Tan, Z. Cao, W. Chu, Q. Wang. Improvement on evaporation-condensation prediction of lee model via a temperature deviation based dynamic correction on evaporation coefficient. Case Stud. Therm. Eng. **48**(103147 (2023). https://doi.org/https://doi.org/10.1016/j.csite.2023.103147
4. H. J. C.Berendsen, D. v. d. Spoel, R. v. Drunen. Gromacs: A message-passing parallel molecular dynamics implementation. Comput. Phys. Commun. **91**(1), 43-56 (1995). https://doi.org/10.1016/0010-4655(95)00042-E
5. J. Wang, R. M. Wolf, J. W. Caldwell, P. A. Kollman, D. A. Case. Development and testing of a general amber force field. J. Comput. Chem. **25**(9), 1157-1174 (2004). https://doi.org/10.1002/jcc.20035
6. W. Humphrey, A. Dalke, K. Schulten. Vmd: Visual molecular dynamics. J. Mol. Graph. **14**(1), 33-38 (1996). https://doi.org/10.1016/0263-7855(96)00018-5
7. J. Wang, P. Cieplak, P. A. Kollman. How well does a restrained electrostatic potential (resp) model perform in calculating conformational energies of organic and biological molecules? J. Comput. Chem. **21**(12), 1049-1074 (2000). https://doi.org/10.1002/1096-987X(200009)21:12<1049::AID-JCC3>3.0.CO;2-F
8. U. Essmann, L. Perera, M. L. Berkowitz, T. Darden, H. Lee, L. G. Pedersen. A smooth particle mesh ewald method. J. Chem. Phys. **103**(19), 8577-8593 (1995). <https://doi.org/10.1063/1.470117>
9. P. E. Blöchl. Projector augmented-wave method. Phys. Rev. B. **50**(24), 17953-17979 (1994). https://doi.org/10.1103/PhysRevB.50.17953
10. G. Kresse, J. Furthmüller. Efficient iterative schemes for ab initio total-energy calculations using a plane-wave basis set. Phys. Rev. B. **54**(16), 11169-11186 (1996). <https://doi.org/10.1103/PhysRevB.54.11169>
11. J. P. Perdew, K. Burke, M. Ernzerhof. Generalized gradient approximation made simple. Phys. Rev. Lett. **77**(18), 3865-3868 (1996). <https://doi.org/10.1103/PhysRevLett.77.3865>
12. S. L. Dudarev, G. A. Botton, S. Y. Savrasov, C. J. Humphreys, A. P. Sutton. Electron-energy-loss spectra and the structural stability of nickel oxide: An LSDA+U study. Phys. Rev. B. **57**(3), 1505-1509 (1998). <https://doi.org/10.1103/PhysRevB.57.1505>
13. S. Grimme, J. Antony, S. Ehrlich, H. Krieg. A consistent and accurate ab initio parametrization of density functional dispersion correction (DFT-D) for the 94 elements h-pu. J. Chem. Phys. **132**(15), (2010). https://doi.org/10.1063/1.3382344
14. H. S. Kang, J. W. Zou, Y. Liu, L. Ma, J. R. Feng, Z. Y. Yu, X. B. Chen, S. J. Ding, L. Zhou, Q. Q. Wang. Synergistic effect of photothermal conversion in Mxene/Au@Cu_2−x_S hybrids for efficient solar water evaporation. Adv. Funct. Mater. **33**(44), 2303911 (2023). https://doi.org/10.1002/adfm.202303911
15. S. Chaule, J. Hwang, S. J. Ha, J. Kang, J. C. Yoon, J. H. Jang. Rational design of a high performance and robust solar evaporator via 3D‐printing technology. Adv. Mater. **33**(38), 2102649 (2021). https://doi.org/10.1002/adma.202102649
16. J. Hu, M. M. Pazuki, R. Li, M. Salimi, H. Cai, Y. Peng, Z. Liu, T. Zhao, M. Amidpour, Y. Wei, Z. Chen. Biomimetic design of breathable 2d photothermal fabric with three-layered structure for efficient four-plane evaporation of seawater. Adv. Mater. **37**(14), 2420482 (2025). https://doi.org/10.1002/adma.202420482
17. X. Wang, Q. Liu, S. Wu, B. Xu, H. Xu. Multilayer polypyrrole nanosheets with self-organized surface structures for flexible and efficient solar-thermal energy conversion. Adv. Mater. **31**(19), 1807716 (2019). https://doi.org/10.1002/adma.201807716
18. M. Zou, Y. Zhang, Z. Cai, C. Li, Z. Sun, C. Yu, Z. Dong, L. Wu, Y. Song. 3D printing a biomimetic bridge‐arch solar evaporator for eliminating salt accumulation with desalination and agricultural applications. Adv. Mater. **33**(34), 2102443 (2021). https://doi.org/10.1002/adma.202102443
19. C. Gao, Y. Li, L. Lan, Q. Wang, B. Zhou, Y. Chen, J. Li, J. Guo, J. Mao. Bioinspired asymmetric polypyrrole membranes with enhanced photothermal conversion for highly efficient solar evaporation. Adv. Sci. **11**(6), (2023). https://doi.org/10.1002/advs.202306833
20. H. Liu, Y. Liu, L. Wang, X. Qin, J. Yu. Nanofiber based origami evaporator for multifunctional and omnidirectional solar steam generation. Carbon **177**, 199-206 (2021). https://doi.org/10.1016/j.carbon.2021.02.081
21. J. Han, Z. Dong, L. Hao, J. Gong, Q. Zhao. Poly(ionic liquid)-crosslinked graphene oxide/carbon nanotube membranes as efficient solar steam generators. Green Energy Environ. **8**(1), 151-162 (2023). https://doi.org/10.1016/j.gee.2021.03.010
22. Q. Xia, Y. Pan, B. Liu, X. Zhang, E. Li, T. Shen, S. Li, N. Xu, J. Ding, C. Wang, C. D. Vecitis, G. Gao. Solar-driven abnormal evaporation of nanoconfined water. Sci. Adv. **10**(22), eadj3760 (2024). https://doi.org/10.1126/sciadv.adj3760
23. Z. Li, X. Zhang, M. Xue, M. Wei, S. Chen, Q. Lu, E. Guo, X. Han, C. Si. Enhanced peroxymonosulfate activation in organic degradation by modulating cationic doping of Ba_x_Sr_1-x_Co_y_Fe_1-y_O_3-δ_ perovskites: DFT calculations and mechanism study. Appl. Catal. B Environ. Energy **377**, 125502 (2025). https://doi.org/10.1016/j.apcatb.2025.125502
24. X. Guo, H. Zhang, Y. Yao, C. Xiao, X. Yan, K. Chen, J. Qi, Y. Zhou, Z. Zhu, X. Sun, J. Li. Derivatives of two-dimensional Mxene-MOFs heterostructure for boosting peroxymonosulfate activation: Enhanced performance and synergistic mechanism. Appl. Catal. B Environ. Energy **323**, 122136 (2023). https://doi.org/10.1016/j.apcatb.2022.122136
25. X. Guo, Q. Zhang, H. He, A. Cai, S. Xi, J. Du, F. Zhang, X. Fan, W. Peng, Y. Li. Wastewater flocculation substrate derived three-dimensional ordered macroporous Co single-atom catalyst for singlet oxygen-dominated peroxymonosulfate activation. Appl. Catal. B Environ. Energy **335**, 122886 (2023). https://doi.org/10.1016/j.apcatb.2023.122886
26. Y.-L. He, C.-S. He, L.-D. Lai, P. Zhou, H. Zhang, L.-L. Li, Z.-K. Xiong, Y. Mu, Z.-C. Pan, G. Yao, B. Lai. Activating peroxymonosulfate by N and O co-doped porous carbon for efficient BPA degradation: A re-visit to the removal mechanism and the effects of surface unpaired electrons. Appl. Catal. B Environ. Energy **314**, 121390 (2022). https://doi.org/10.1016/j.apcatb.2022.121390
27. Y. Long, Z. Cao, W. Wu, W. Liu, P. Yang, X. Zhan, R. Chen, D. Liu, W. Huang. Rational modulation of fe single-atom electronic structure in a Fe-N_2_B_4_ configuration for preferential ^1^O_2_ generation in fenton-like reactions. Appl. Catal. B Environ. Energy **344**, 123643 (2024). https://doi.org/10.1016/j.apcatb.2023.123643
28. R. Li, M. Wu, H. Ma, Y. Zhu, H. Zhang, Q. Chen, C. Zhang, Y. Wei. A single component, single layer flexile foam evaporator with the higher efficiency for water generation. Adv. Mater. **36**(29), 2402016 (2024). https://doi.org/10.1002/adma.202402016
29. L. Tian, L. Han, F. Wang, H. Shen, Q. Li, L. Zhu, S. Chen. Dynamic water microskin induced by photothermally responsive interpenetrating hydrogel networks for high-performance light‐tracking water evaporation. Adv. Energy Mater. **15**(10), 2404117 (2025). https://doi.org/10.1002/aenm.202404117
30. Z. Wang, M. Hu, L. Zhu, J. Zhou, F. He, Y. Liu, Y. Li, Y. Li, Z. Lin, F. Caruso. Cracked metal-phenolic networks with durable confinement capillarity for enhanced solar desalination. Adv. Mater. **37**(33), 2503896 (2025). https://doi.org/10.1002/adma.202503896
31. P. Cheng, M. Klingenhof, H. Honig, L. Zhang, P. Strasser, P. Schaaf, D. Lei, D. Wang. Synergizing plasmonic local heating and 3D nanostructures to boost the solar-to-vapor efficiency beyond 100%. Adv. Mater. **37**(5), 2415655 (2024). https://doi.org/10.1002/adma.202415655
32. C. Ling, Z. Zhang, T. Dong, C. Zhu, Y. Xue, J. Han, F. Liu. Constructing low-temperature-resistant advanced oxidation process by hollow porous carbon-supported single-atom Fe catalyst for efficient cold-water decontamination: Combined kinetic and thermodynamic optimization. Appl. Catal. B Environ. Energy **372**, 125330 (2025). https://doi.org/10.1016/j.apcatb.2025.125330
33. F.-Z. Jiao, J. Wu, T. Zhang, R.-J. Pan, Z.-H. Wang, Z.-Z. Yu, J. Qu. Simultaneous solar-thermal desalination and catalytic degradation of wastewater containing both salt ions and organic contaminants. ACS Appl. Mater. Interfaces **15**(34), 41007-41018 (2023). https://doi.org/10.1021/acsami.3c09346
34. L. Cui, C. Ma, P. Wang, H. Che, H. Xu, Y. Ao. Rationally constructing a 3D bifunctional solar evaporator for high-performance water evaporation coupled with pollutants degradation. Appl. Catal. B Environ. Energy **337**, 122988 (2023). https://doi.org/10.1016/j.apcatb.2023.122988
35. T. Zhang, J. Qu, J. Wu, F. Z. Jiao, C. Li, F. L. Gao, J. Liu, Z. Z. Yu, X. Li. All-in-one self-floating wood-based solar-thermal evaporators for simultaneous solar steam generation and catalytic degradation. Adv. Funct. Mater. **34**(39), 2403505 (2024). https://doi.org/10.1002/adfm.202403505
36. R.-J. Pan, J. Wu, J. Qu, T. Zhang, F.-Z. Jiao, M. Zhao, M.-Y. Han, X. Li, Z.-Z. Yu. Peak-like three-dimensional CoFe_2_O_4_/carbon nanotube decorated bamboo fabrics for simultaneous solar-thermal evaporation of water and photocatalytic degradation of bisphenol A. J. Mater. Sci. Technol. **179**, 40-49 (2024). https://doi.org/10.1016/j.jmst.2023.08.045
